# Novel hypoxia-related gene signature for predicting prognoses that correlate with the tumor immune microenvironment in NSCLC

**DOI:** 10.3389/fgene.2023.1115308

**Published:** 2023-04-06

**Authors:** Zhaojin Li, Yu Cui, Shupeng Zhang, Jie Xu, Jianping Shao, Hekai Chen, Jingzhao Chen, Shun Wang, Meizhai Zeng, Hao Zhang, Siqian Lu, Zhi Rong Qian, Guoqiang Xing

**Affiliations:** ^1^ Department of General Surgery, Tianjin Fifth Central Hospital, Tianjin, China; ^2^ Beidou Precision Medicine Institute, Guangzhou, China

**Keywords:** NSCLC, hypoxia, prognosis, immune microenvironment, immunotherapy

## Abstract

**Background:** Intratumoral hypoxia is widely associated with the development of malignancy, treatment resistance, and worse prognoses. The global influence of hypoxia-related genes (HRGs) on prognostic significance, tumor microenvironment characteristics, and therapeutic response is unclear in patients with non-small cell lung cancer (NSCLC).

**Method:** RNA-seq and clinical data for NSCLC patients were derived from The Cancer Genome Atlas (TCGA) database, and a group of HRGs was obtained from the MSigDB. The differentially expressed HRGs were determined using the limma package; prognostic HRGs were identified via univariate Cox regression. Using the least absolute shrinkage and selection operator (LASSO) and multivariate Cox regression, an optimized prognostic model consisting of nine HRGs was constructed. The prognostic model’s capacity was evaluated by Kaplan‒Meier survival curve analysis and receiver operating characteristic (ROC) curve analysis in the TCGA (training set) and GEO (validation set) cohorts. Moreover, a potential biological pathway and immune infiltration differences were explained.

**Results:** A prognostic model containing nine HRGs (STC2, ALDOA, MIF, LDHA, EXT1, PGM2, ENO3, INHA, and RORA) was developed. NSCLC patients were separated into two risk categories according to the risk score generated by the hypoxia model. The model-based risk score had better predictive power than the clinicopathological method. Patients in the high-risk category had poor recurrence-free survival in the TCGA (HR: 1.426; 95% CI: 0.997–2.042; *p* = 0.046) and GEO (HR: 2.4; 95% CI: 1.7–3.2; *p* < 0.0001) cohorts. The overall survival of the high-risk category was also inferior to that of the low-risk category in the TCGA (HR: 1.8; 95% CI: 1.5–2.2; *p* < 0.0001) and GEO (HR: 1.8; 95% CI: 1.4–2.3; *p* < 0.0001) cohorts. Additionally, we discovered a notable distinction in the enrichment of immune-related pathways, immune cell abundance, and immune checkpoint gene expression between the two subcategories.

**Conclusion:** The proposed 9-HRG signature is a promising indicator for predicting NSCLC patient prognosis and may be potentially applicable in checkpoint therapy efficiency prediction.

## 1 Introduction

Lung cancer ranks highly among all malignancies in its prevalence and mortality. Non-small cell lung cancer (NSCLC) accounts for approximately 85% of all lung cancer diagnoses (Siegel et al., 2023). Although great advances have been made in the research and treatment of NSCLC, the majority of patients are diagnosed at an advanced stage, and the efficacy of NSCLC treatment remains unsatisfactory. NSCLC diagnosis and prognosis are currently determined by tumor stage and histopathological assessment (Cuyun Carter et al., 2014; Wood et al., 2022). Unfortunately, the accuracy of these standard diagnostic procedures is insufficient, and the resulting illness outcomes in patients with a uniform clinical diagnosis can also be inconsistent (Balachandran et al., 2015). Consequently, the development of a unique, accurate, and sensitive signature for early NSCLC detection and prognostic prediction is urgently needed.

Because the demand for oxygen in the tumor is greater than the body supplies, hypoxia has been discovered to accelerate tumor development with the induction of a hypoxic tumor context (Petrova et al., 2018). The hypoxic environment within tumors is one of their most important hallmarks. Tumor formation and incidence are frequently accompanied by a variety of adaptive changes involving transcription factor activation, cell proliferation, motility, apoptosis, and other signaling pathways through which hypoxia might enhance tumor aggressiveness and medication resistance (Gilkes et al., 2014; Muz et al., 2015; Rankin et al., 2016; Nobre et al., 2018; Jing et al., 2019).

With the popularization of transcriptome sequencing technology, an increasing number of studies have shown that hypoxic tumor microenvironments are associated with worse clinical presentation and prognosis (Magnon et al., 2007; D'Ignazio et al., 2017). Previous studies have demonstrated that hypoxia-related genes (HRGs) are linked to prognosis in LUAD patients with disease stages I–II (Sun et al., 2020). However, clinical outcomes, oncogenic pathways, and treatment responses remain unclear in NSCLC patients.

Prior studies have verified the critical roles of hypoxia in promoting immunological escape and tumor immune suppression. For example, hypoxia stimulates the recruitment of immunosuppressive cytokines and suppressive cells, consequently hampering immune effector cells and prompting immunological escape (Qian et al., 2019). Because the codependency between the immunological state and hypoxia in the tumor microenvironment could lead to alterations in immune activity, treatment response, and prognosis in NSCLC, an integrated analysis of the immunological state and hypoxia might have promising prognostic utility and provide supplementary knowledge to facilitate the development of transformational studies on and treatment strategies for NSCLC.

We thus extracted data from multiple independent NSCLC cohorts to screen candidate HRGs and established a related signature, with the aim of determining its capacity to predict clinical outcomes, TME features, and therapeutic efficacy in NSCLC patients.

## 2 Methods

**FIGURE 1:**
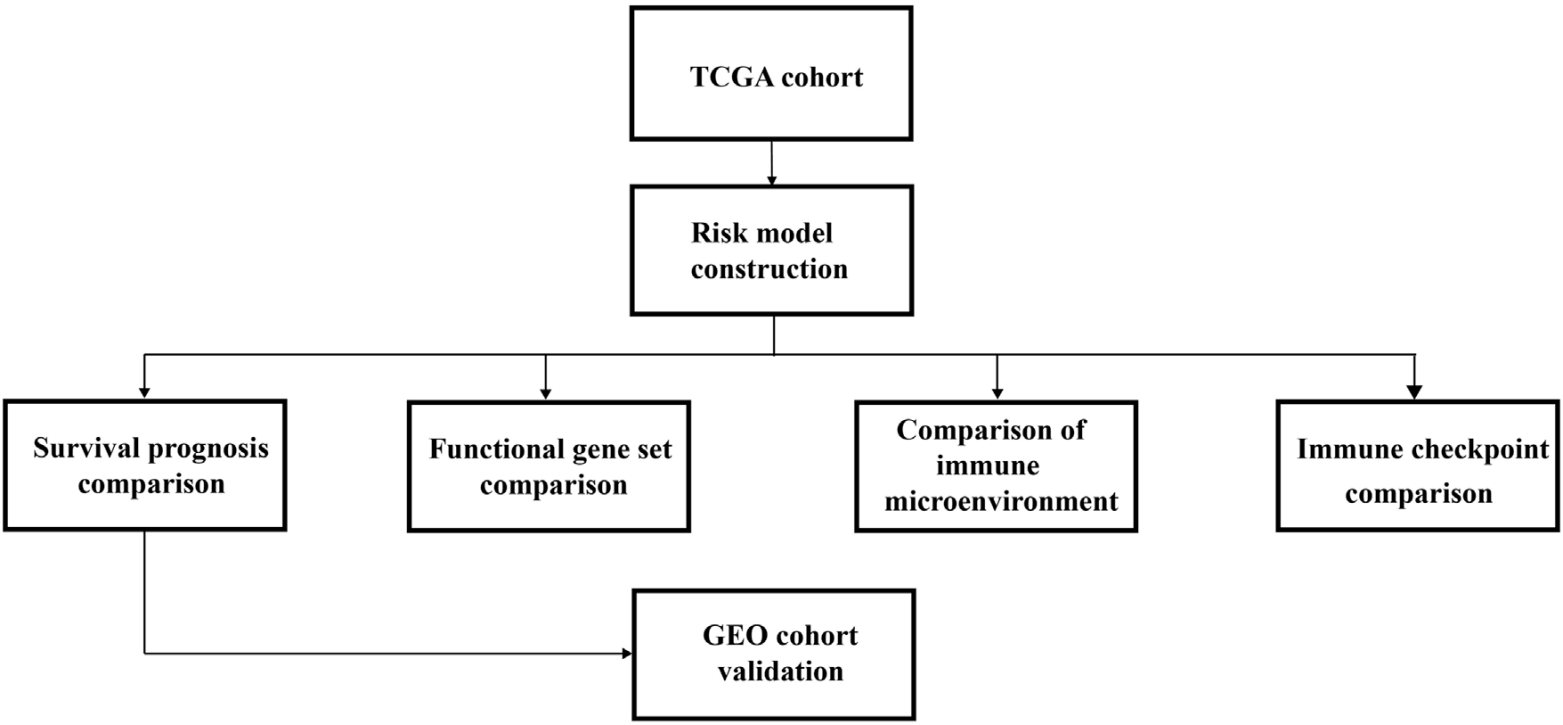
Diagrammatic representation of the analytical process used in this study.

The overall research strategy, including the building and verification of the HRG risk model, is depicted in Figure 1.

### 2.1 Data preparation and processing

From the UCSC Xena database, NSCLC clinical data and transcriptome expression profiles for LUAD and LUSC patients in The Cancer Genome Atlas (TCGA) were downloaded. A total of 1,011 tumor and 108 normal samples were employed for HRG expression differential analysis and as a training set for the construction of the risk score model. All scores indicating the degree of hypoxia in the tumor, such as the Winter, Buffa, and Ragnum hypoxia scores (Winter et al., 2007; Buffa et al., 2010; Ragnum et al., 2015), were obtained from TCGA. The higher these scores, the more hypoxic is the tumor.

For external validation, four microarray datasets together with detailed clinical characteristics were retrieved from the Gene Expression Omnibus (GEO) (GSE30219, GSE31210, GSE37745, and GSE50081) using the GEOquery R package. A total of 628 tumor samples were acquired, and the clinical characteristics are listed in Supplementary Table S1. Batch effects were eliminated using the sva package (Leek et al., 2012), and the scale function was used to standardize the expression value before model construction and validation.

### 2.2 Identification of differentially expressed HRGs

A total of 190 of 200 HRGs and available data were retrieved from the MSigDB Hallmark database (Supplementary Table S2), and the expression of these genes was extracted from integrated datasets (Liberzon et al., 2015). Differential analysis between tumor and normal samples of 190 HRGs was performed using the limma package in the TCGA database. Genes with *p* values less than 0.05 were defined as differentially expressed HRGs (DEHRGs). Volcano mapping was then performed using the ggplot2 package.

### 2.3 Building and verifying an HRG-based risk model

We conducted univariate Cox regression analysis based on overall survival to determine prognostic DEHRGs (*p* < 0.05). The DEHRGs related to OS were identified by least absolute shrinkage and selection operator (LASSO) regression analysis using the glmnet package to shrink the gene set associated with prognosis. To achieve the optimal model, we combined the remaining genes step by step and tested performance. Ultimately, nine genes were selected, and a multivariate Cox regression analysis was adopted to build a prognostic risk model. The algorithm used to determine the patient’s risk level was as follows:

$$\begin{array}{l} Risk\;score=0.10515\times {STC2}_{\exp}+0.0234\times {ALDOA}_{\exp}+0.02207\\ \times {MIF}_{\exp}+0.13426\times {LDHA}_{\exp}+0.09928\times {EXT1}_{\exp}-0.01151\\ \times {PGM2}_{\exp}-0.1475\times {ENO3}_{\exp}-0.16294\times {INHA}_{\exp}-0.14694\times {RORA}_{\exp} \end{array}$$

A risk score was then obtained for all patients. Patients were separated into two risk categories by using the median risk score of patients.

To determine the predictive ability of this model for tumor recurrence, recurrence-free survival was compared between two risk categories using stage I and II cases in the TCGA and GEO databases.

Overall survival (OS) was also compared between the two risk categories using all patients. The survivalROC R package was employed to plot the receiver operating characteristic (ROC) curves of 1-, 3-, and 5-year OS in the training cohort, and the same method was used in the validation cohort.

### 2.4 Independent determination of risk models and construction of clinical nomograms

Multivariate Cox regression analysis was applied to construct an independent prognostic model. A nomogram was used to predict patient survival rates, and the performance was verified by drawing calibration curves. ROC curves and the concordance index were introduced to detect the reliability of the HRG model. Clinical features were incorporated into multivariate Cox regression to determine whether the risk model was an independent indicator of prognostic prediction.

### 2.5 Functional enrichment analysis

The gene sets of the Kyoto Encyclopedia of Genes and Genomes and the Hallmark gene sets were downloaded from MSigDB. The enrichment scores of individual patients were computed with the GSVA R package. Using the limma package, the enrichment score was then compared between two TCGA risk categories, and the difference in HRG expression between the two categories was also analyzed. A cluster heatmap was used to show the top 30 genes with the most significant *p* values.

### 2.6 Immunological features of the tumor microenvironment

Immunomodulators, a group of immunoregulatory genes containing antigen-presenting factors, ligands, and receptors, play crucial roles in cancer immunotherapy, and the expression of HRGs and immunomodulators was compared with the Spearman correlation method.

In addition, the differences in their infiltration abundance in the tumor microenvironment affected the patients’ outcomes and the efficiency of immunotherapy. Therefore, the ssGSEA algorithm was employed to compute immune cell abundance in individual patients. Using the Spearman correlation method, the correlation between the hypoxia-related model and immune cells was analyzed, and, finally, the difference in immune cell abundance between the two risk categories was assessed.

Immunomodulator-related genes and immune cell-type marker genes were obtained from the TCGA immune response working group (Charoentong et al., 2017; Thorsson et al., 2018). The immunological score was computed with the estimate package, and we compared the difference between the two risk categories.

### 2.7 Statistical analysis

Kaplan‒Meier curves and the log-rank test were used to compare the survival rate between various subcategories. The independent prognostic significance of the clinical features in OS was detected by univariate and multivariate Cox proportional hazard regression analyses. The prognostic prediction significance of the risk models for 1/3/5-year OS was evaluated by ROC curves and area under the curve (AUC) values. Differences in TMN stage and clinicopathologic stage composition between the two risk categories of the TCGA cohort were analyzed by Fisher’s test. The correlation analysis of HRGs with immune cell abundance and with immune regulator-related genes was performed using Spearman’s correlation analysis. The Wilcoxon test was applied to compare immune cell infiltration. *p* values less than 0.05 were defined as significant. R 3.6.1 was used to conduct all the analyses.

## 3 Results

### 3.1 Differential expression analysis of HRGs in the training cohort

Genes that are expressed normally in both tumor and normal tissues do not typically play a significant role in the development and progression of tumors. Therefore, it is necessary to exclude such genes from the analysis beforehand.

**FIGURE 2:**
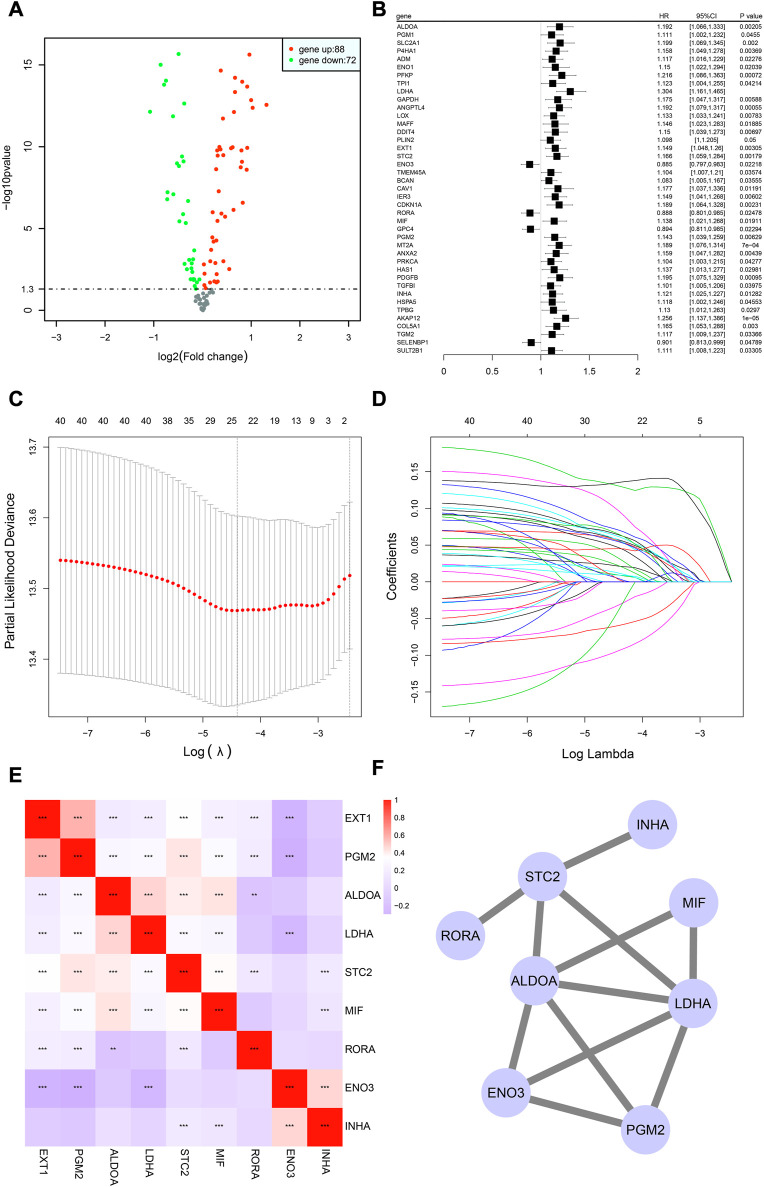
Building of the HRG model in the TCGA-NSCLC cohort. **(A)** Volcano graphic showing how HRG expression differs in tumor and normal samples based on both fold change and statistical significance. **(B)** Results of univariate Cox regression analysis presented in a forest plot, with gene name in the first column and box plot depicting the hazard ratio (HR) and its corresponding 95% confidence interval in the second column. **(C,D)** LASSO analysis for estimating the number of contributing components. **(E)** Correlation analysis of HRG expression in the risk model using Spearman’s correlation. *p < 0.05; **p < 0.01; ***p < 0.001. **(F)** Results of protein interaction analysis of the HRG model.

Differential analysis between 1,011 tumor and 108 normal samples on 190 HRGs was performed, and 160 genes were significantly differentially expressed (Supplementary Table S3) in the TCGA cohort. Of these, 72 genes were downregulated in tumor tissues, while 88 were upregulated. The differentially expressed volcano map is shown in Figure 2A.

The analysis revealed that a majority of HRGs showed significant differences in expression, highlighting the importance of the hypoxia pathway as a mechanism underlying tumor growth and metastasis.

### 3.2 Building of the HRG model in the training cohort

Although there may be aberrant gene expression, it may not necessarily affect a patient’s survival status or time. Thus, the identification of hypoxia-related genes that are linked to prognosis is crucial.

**FIGURE 3:**
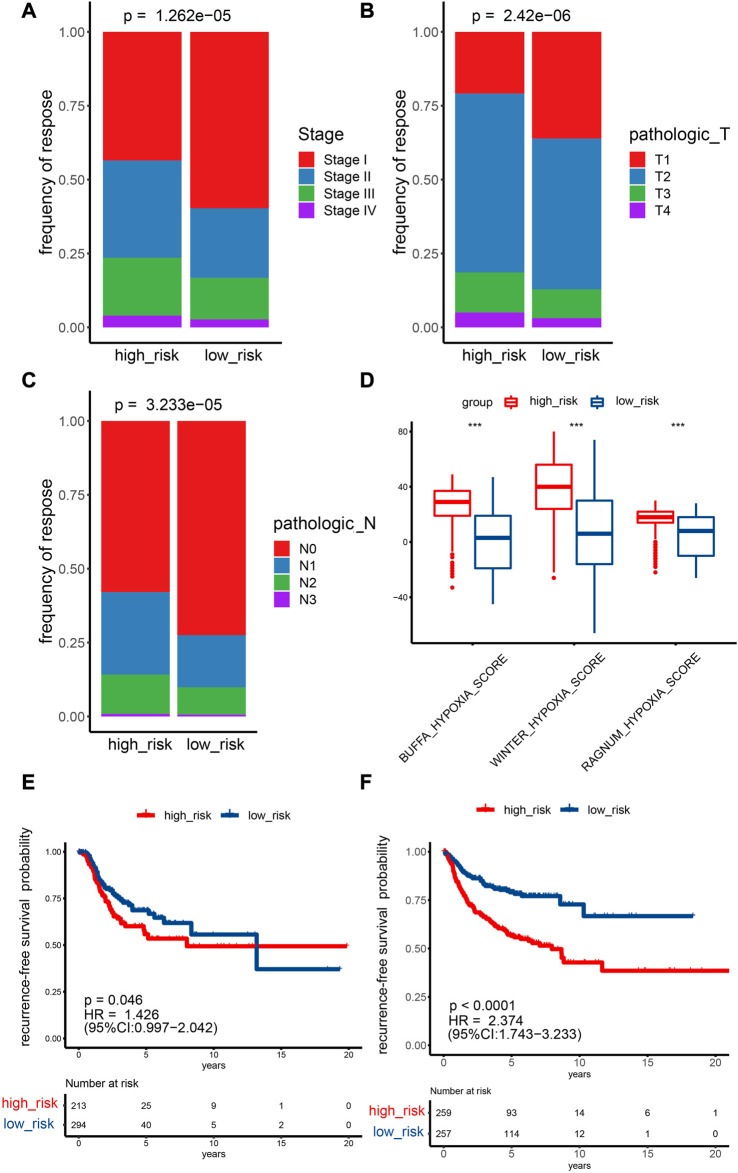
Prediction ability of the HRG model for the recurrence and stage of NSCLC patients. **(A–C)** TCGA cohort Fisher test outcomes for T and N stages and pathological stages comparing two risk categories. **(D)** Analyzing the differences between two risk categories on three known hypoxia scores. *p < 0.05; **p < 0.01; ***p < 0.001. **(E,F)** Recurrence survival curves for two risk categories. E: TCGA cohort, F: GEO cohort.

In total, 990 NSCLC cases in the TCGA cohort with detailed OS information were identified. To further identify prognosis-related DEHRGs, 160 DEHRGs were analyzed using univariate Cox regression analysis, and 41 genes were significantly correlated with prognosis (Supplementary Table S4). A forest map was used to display the prognosis-related HRGs’ hazard ratios and the matching confidence intervals (CIs), which demonstrated that the majority of candidates were at risk (Figure 2B). To ensure the stability and feasibility of the model, LASSO regression analysis was performed with the optimum *λ* value to reduce the scope of candidates, and 25 DEHRGs were identified (Figures 2C, D). Finally, by performing multivariate Cox regression, nine genes (STC2, ALDOA, MIF, LDHA, EXT1, PGM2, ENO3, INHA, and RORA) were selected to construct a risk prognostic model. In the TCGA training cohort, patients were split into two risk categories based on the median risk score. Notably, compared to patients in the low-risk category, those in the high-risk category were more likely to experience relapse (HR: 1.426; 95% CI: 0.997–2.042; *p* = 0.046; Figure 3E).

A linear regression analysis was conducted to determine the interaction of these nine genes; the results indicated that 25 candidates exhibited high correlation, such as ALDOA and LDHA, ALDOA and STC2, ALDOA and MIF, and ENO3 and EXT1 (Figure 2E). In addition, protein interaction analysis was performed on these nine genes using the STRING online database and visualized by Cytoscape. We observed that ALDOA, LDHA, ENO3, and PGR2 strongly interacted with multiple genes—in the end, these four candidates were regarded as the core genes (Figure 2F).

Fisher’s test showed that the percentage of stage I samples, T1 samples, and N0 samples in the low-risk category (stage: 59.7%; T: 36.1%; N: 72.5%) was greater than the percentage in the high-risk category (stage: 43.5%; T: 20.9%; N: 57.9%) (Figures 3A–C). The difference in M0 staging in the low-risk category was not significant in comparison with the low-risk category (Supplementary Figure S1). A higher risk score indicates higher expression levels of most HRGs, which are associated with a more advanced disease stage, a larger tumor size, and increased lymph node involvement. One possible explanation for the higher proportion of stage I, T1, and N0 samples in the low-risk category compared with the high-risk category is that HRGs are expressed more strongly in later-stage and more invasive tumors, which may lead to higher risk scores and worse prognoses. In addition, the lack of a significant difference in M0 staging between the low- and high-risk categories may be due to distant metastasis being less affected by hypoxia than local tumor growth and invasion. The Buffa, Winter, and Ragnum hypoxia scores in the high-risk category were greater than those in the low-risk category (Figure 3D), implying that the established model could effectively evaluate the degree of hypoxia.

**FIGURE 4:**
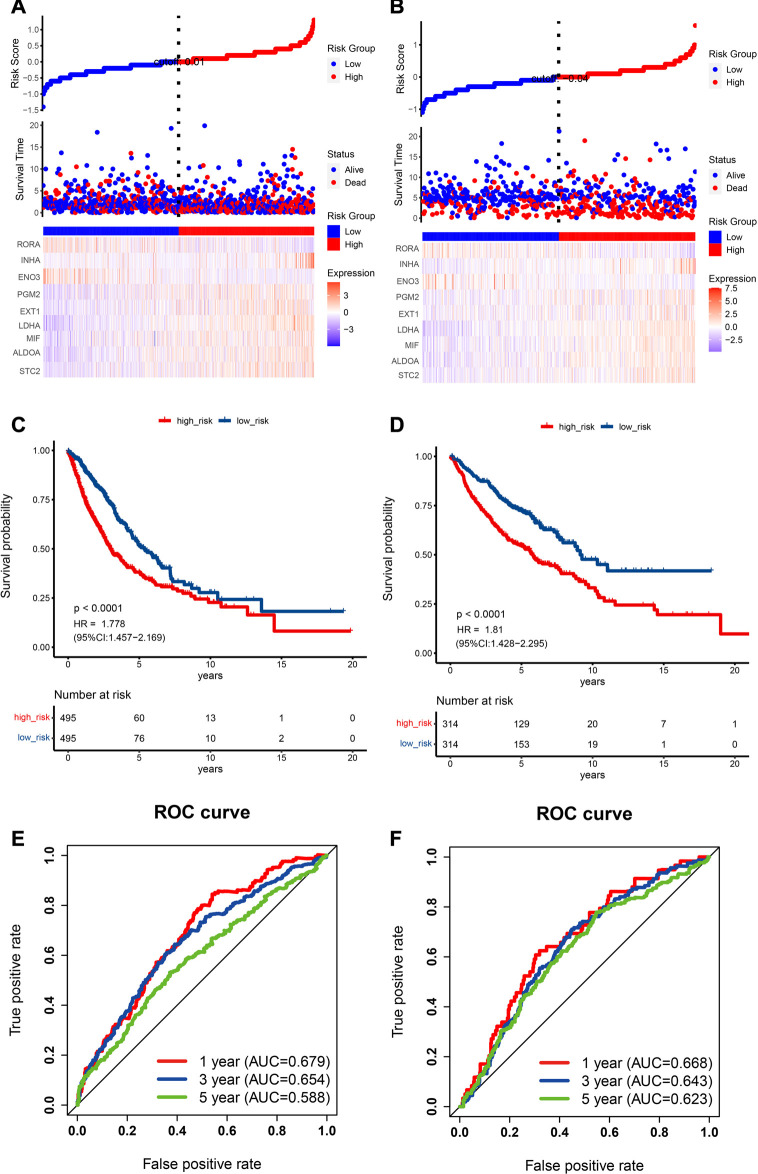
Prognostic data analysis from the training and validation cohorts as well as verification of the model’s precision. **(A,B)** The risk score curve is displayed in the top part. The distribution of the risk score, survival duration, and patient status are presented in the middle part. A heatmap of HRGs in the classifier is shown in the bottom part. **(A)** TCGA cohort, **(B)** GEO cohort. **(C,D)** NSCLC patient Kaplan‒Meier survival curve comparing two risk categories. **(C)** TCGA cohort, **(D)** GEO cohort. **(E,F)** ROC curve in the TCGA cohort **(E)** and GEO cohort **(F)**.

Moreover, the scatter plot of OS status showed that, in comparison with low-risk category patients, more deaths were observed in the high-risk category (Figure 4A). More importantly, high-risk category patients (n = 495) had a worse prognosis than low-risk patients (n = 495) (*p* < 0.0001; HR: 1.778; 95% CI: 1.457–2.169; Figure 4C). To accurately evaluate the prognostic performance of the nine-gene signature, ROC analysis was conducted using the 1-, 3-, and 5-year cutoff values of OS—the AUC values were 0.679, 0.654, and 0.588, respectively, indicating that our prognostic model had good predictive performance in patients with NSCLC (Figure 4E).

Early-stage cancer patients are more highly represented in the low-risk category based on HRGs than in the high-risk category. Thus, when we specifically only analyzed early-stage patients, we discovered that significant differences in overall survival still existed between high- and low-risk categories in early-stage cancer prognostic indicators (Supplementary Figure S2). This finding suggests that risk stratification based on HRGs can still be used to effectively predict the overall survival of NSCLC patients, even when analyzing only the subset of patients in the early stages of the disease.

To summarize, a model based on the expression of HRGs that are prognostically relevant can effectively assess the extent of hypoxia, exhibit a close correlation with clinical staging, and reveal significant differences in recurrence, survival, and overall survival between high- and low-risk categories.

### 3.3 External validation of the HRG prognostic model

External validation plays a crucial role in improving the reliability and reproducibility of scientific research. By confirming the generalizability and applicability of research findings, external validation can help to identify and correct potential problems and limitations in study design and methodology.

To evaluate the prognostic capability of this novel HRG-based risk model, external cohorts were obtained from the GEO. The risk score was computed *via* the identical formula described before. The patients were separated into two risk categories using the median score. In recurrence-free survival analysis, patients in the high-risk category had a higher tendency to relapse than low-risk patients (HR: 2.374; 95% CI: 1.743–3.233; *p* < 0.0001; Figure 3F).

Scatter plots for death events are displayed in Figure 4B. High-risk category patients showed higher mortality rates than patients in the low-risk category. Additionally, we confirmed the performance of our HRG risk model for OS in the GEO cohort. As expected, a worse OS was indicated by a higher risk score (*p* < 0.0001, HR: 1.81, 95% CI: 1.428–2.295; Figure 4D). Figure 4F shows the 1-, 3-, and 5-year cutoff OS values in the validation dataset; the AUC values were 0.668, 0.643, and 0.623, respectively. ROC analysis further verified the effectiveness and reliability of the HRG risk model for prognostic prediction in NSCLC patients.

In our external dataset validation analysis, we found that our model demonstrated strong predictive performance for both overall survival and recurrence-free survival, indicating that it is both stable and generalizable.

### 3.4 Analysis of independent prognostic factors in the HRG risk model

**FIGURE 5:**
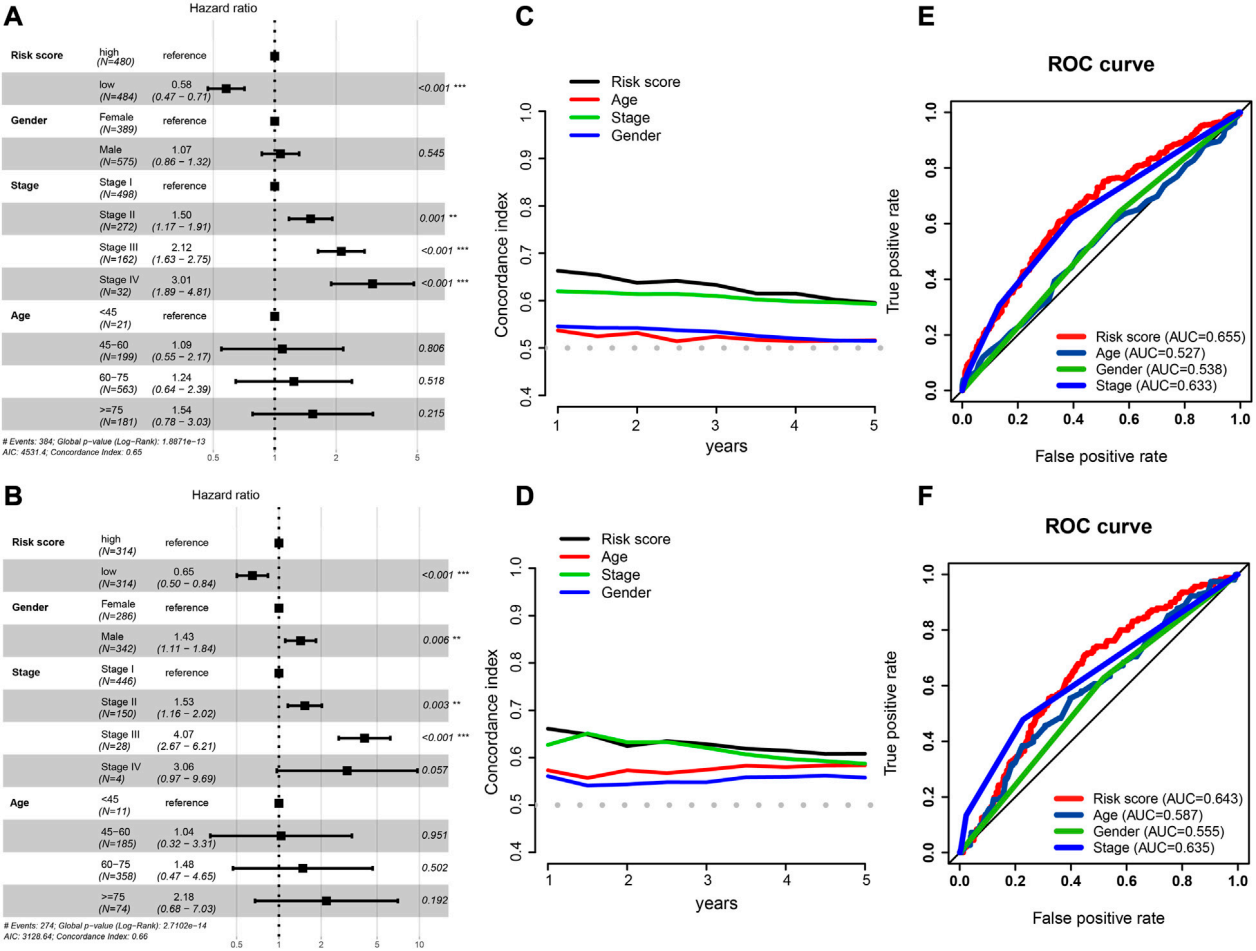
Independent prognostic factor determination and predictive accuracy comparison. **(A,B)** Results of TCGA cohort **(A)** and GEO cohort’s multivariate Cox regression analysis **(B)**. **(C,D)** Concordance index curve of 3 clinical parameters and risk scores for OS time from 1 to 5 years, **(C)** TCGA cohort, **(D)** GEO cohort. **(E,F)** Multi-index ROC curve of the risk score and other clinical parameters for 3-year OS time, TCGA cohort **(E)** and GEO cohort **(F)**.

Patients with complete clinical information were identified to construct risk prognostic models; Table 1 displays the clinical information. Along with risk scores, clinical stage, age, and sex were considered in a multivariate Cox regression analysis to determine independent prognostic markers. According to the outcome, the risk score was an independent prognostic factor in the TCGA (HR: 0.58, 95% CI: 0.47–0.71; Figure 5A) and GEO cohorts (HR: 0.65, 95% CI: 0.50–0.84; Figure 5B).

**TABLE 1:** The clinical pathological information of TGCA and GEO cohorts.

	TCGA cohort (n = 964)	GEO cohort (n = 628)
Gender		
Female	389 (40.4%)	286 (45.5%)
Male	575 (59.6%)	342 (54.5%)
Age (years)		
<45	21 (2.2%)	11 (1.8%)
≥75	181 (18.8%)	74 (11.8%)
45–60	199 (20.6%)	185 (29.5%)
60–75	563 (58.4%)	358 (57.0%)
Overall survival time		
Mean (SD)	2.52 (2.52)	4.78 (3.23)
Median [min, max]	1.78 [0.00274, 19.9]	4.61 [0.0164, 21.3]
Clinical stage		
Stage I	498 (51.7%)	446 (71.0%)
Stage II	272 (28.2%)	150 (23.9%)
Stage III	162 (16.8%)	28 (4.5%)
Stage IV	32 (3.3%)	4 (0.6%)
Recurrence-free survival time		
Mean (SD)	2.66 (2.63)	4.26 (3.29)
Median [min, max]	1.79 [0.0110,19.9]	4.31 [0.160,21.3]
Missing	391 (40.6%)	90 (14.3%)

We also calculated the conformance index (C-index) of these three clinical factors and risk score within 1–5 years of OS time. For the TCGA cohort, the results suggested that the C-index of the risk score was greater than that of other clinical factors (Figure 5C), and that the C-index had a similar numerical approximation between the risk score and clinicopathological stage in the GEO cohort (Figure 5D).

The ROC curve of the risk score was detected with these three clinical factors for the 3-year OS. The AUC value of the risk score was greater than that of the other three clinical characteristics in both cohorts (Figures 5E, F).

Taken together, even when other factors that may affect patient prognoses were taken into account, the risk score had a significant and distinct impact on patient outcomes. It performed better than other clinical parameters in predicting overall survival.

### 3.5 Building and verifying the prognostic nomogram system

To assist clinical practitioners with more accurate predictions of patient survival rates during treatment and management, we constructed a clinical nomogram that serves as an important auxiliary decision-making tool.

**FIGURE 6:**
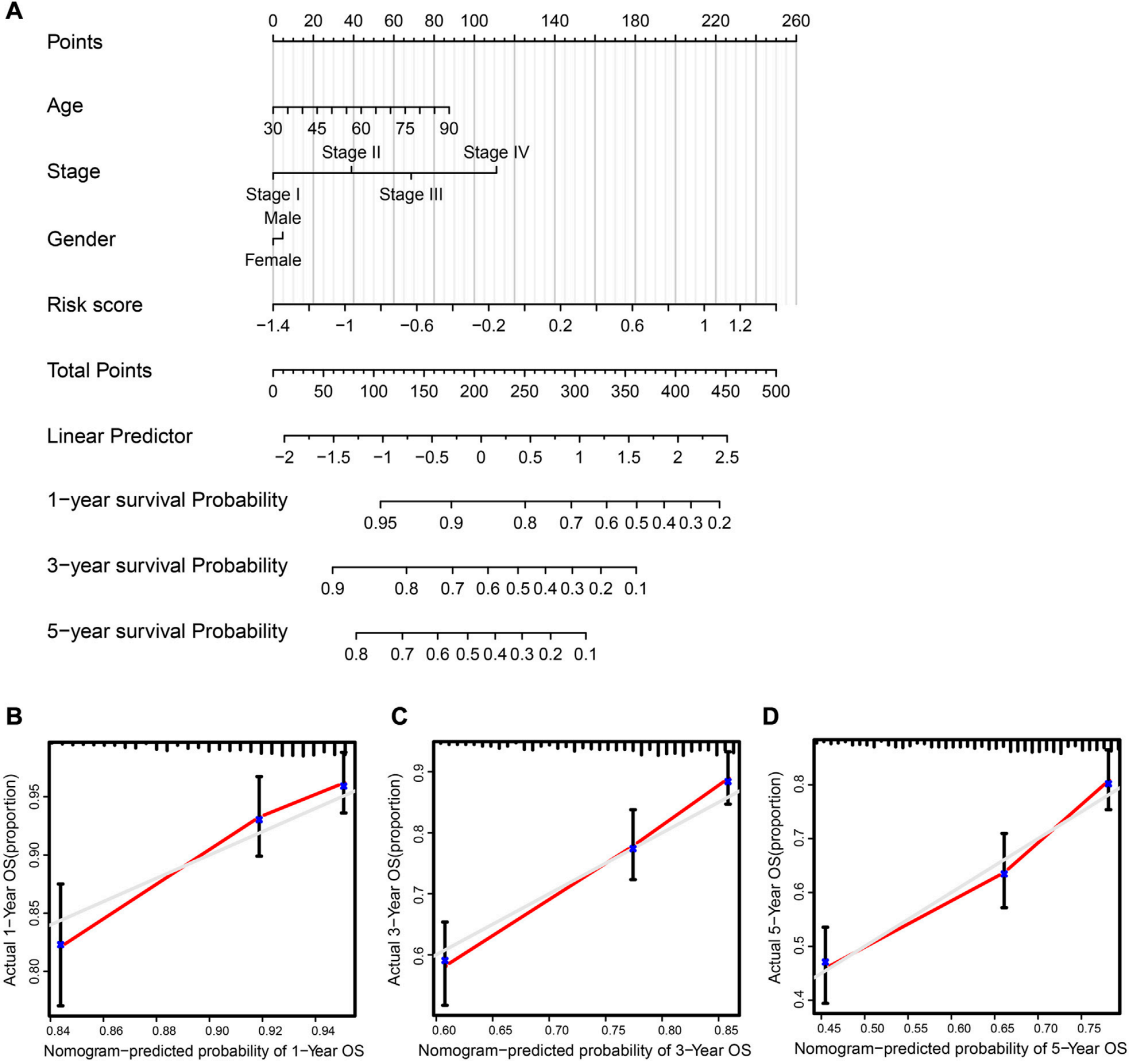
Clinical prognostic nomogram was created and validated. **(A)** Nomogram, taking into account risk score, tumor stage, age, and sex, predicted the likelihood of 1-, 3-, and 5-year OS. **(B–D)** 1-, 3-, and 5-year OS calibration curves; predicted survival probability graphed along the x-axis, whereas actual survival probability is represented along the y-axis.

Using the “rms” and “survival” R packages, we generated a nomogram containing the risk score and valuable clinical parameters predicting 1-, 3-, and 5-year overall survival of NSCLC patients in the TCGA dataset and a calibration plot that analyzed estimated and actual overall survival to assess the effectiveness of the prognostic nomogram (Figure 6A). The calibration plot showed that the nomogram’s estimated survival and actual survival rates were highly correlated (Figures 6B–D). It is possible to evaluate the probability of a patient surviving at a certain time according to the patient’s clinical information and gene expression levels using this clinical prognosis nomogram. In conclusion, this nomogram system for predicting the overall survival of patients based on gene expression and clinical information is reliable.

### 3.6 Biological pathway analysis related to the HRG risk model

Different biological mechanisms often lead to distinct prognostic outcomes in cancer patients. KEGG pathway and cancer-related hallmark gene set analyses were performed to clarify the biological mechanisms that were related to the risk model.

**FIGURE 7:**
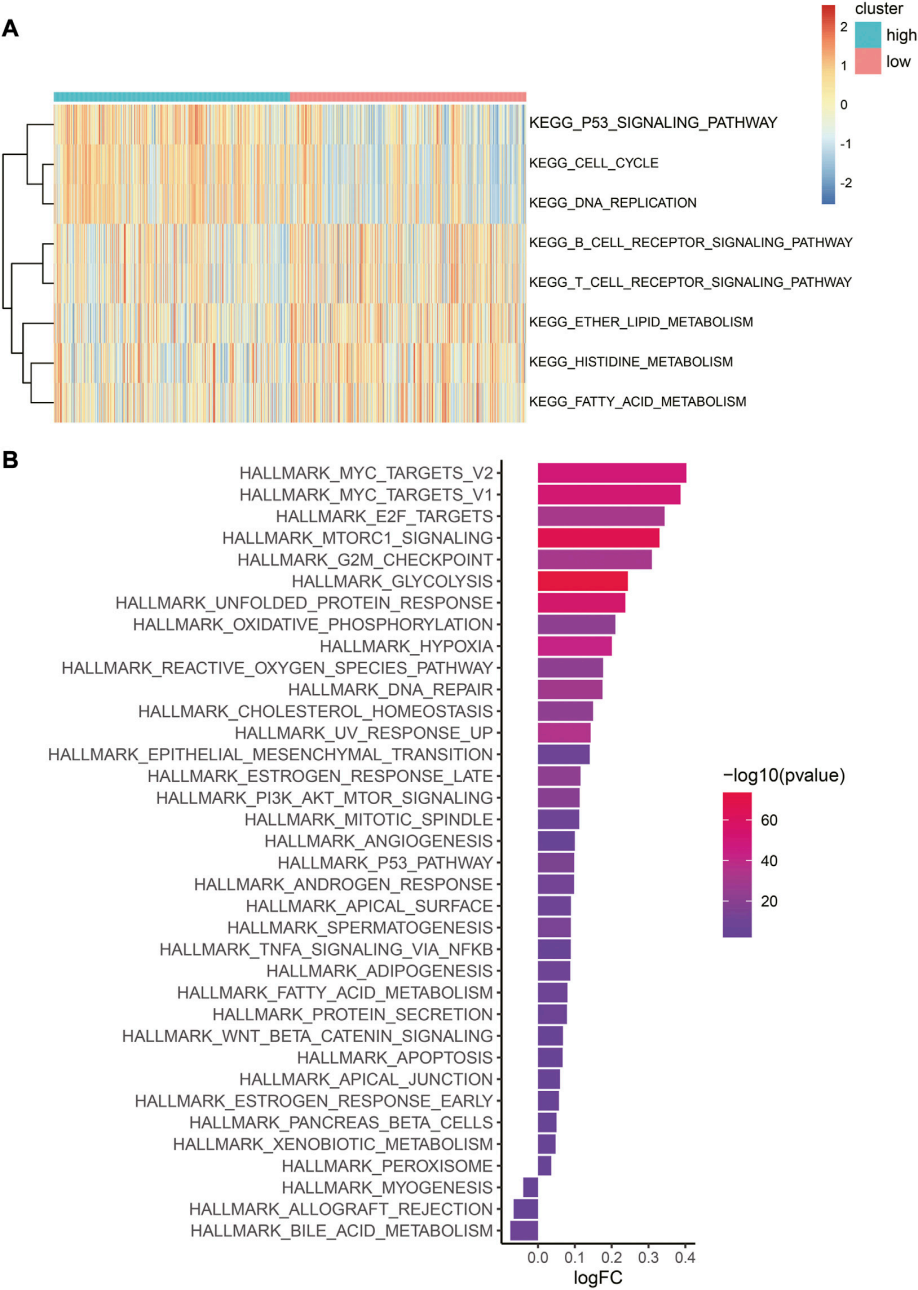
Difference in biological pathways and cancer-related gene sets between the two risk categories. **(A)** Heatmap of GSVA enrichment of the KEGG pathway. Red represents high enrichment scores, while blue represents low enrichment scores. **(B)** Bar graph of hallmark gene set enrichment score. The color indicates the significance of the difference, and the x-axis represents the enrichment score fold change in this hallmark gene set between high- and low-risk categories.

In the low-risk category, several metabolically related pathways, such as histidine, fatty acid, and ether lipid metabolism, were highly enriched (Figure 7A). The metabolism of these amino acids and lipids was reported to be closely associated with tumors (Currie et al., 2013; Li and Zhang, 2016; Bian et al., 2021). Interestingly, immune-related pathways were observed to cluster in the low-risk category, such as the T-cell and B-cell receptor signaling pathways. Conversely, for the high-risk category, cell proliferation-related pathways such as the G2/M checkpoint, E2F targets, and MYC targets were enriched. It is worth noting that hypoxia was also identified in the high-risk category, as anticipated (Figure 7B). The expression heatmap of the 30 most significant DEHRGs between the two risk categories is shown in Supplementary Figure S3. It is evident from the heatmap that there is a significant difference in the expression levels of these genes.

In summary, there are significant mechanistic differences between the low-risk and high-risk categories, with the immune features of the former and proliferation characteristics of the latter being consistent with their respective prognostic outcomes.

### 3.7 Characteristics of the tumor immune microenvironment associated with HRG models

**FIGURE 8:**
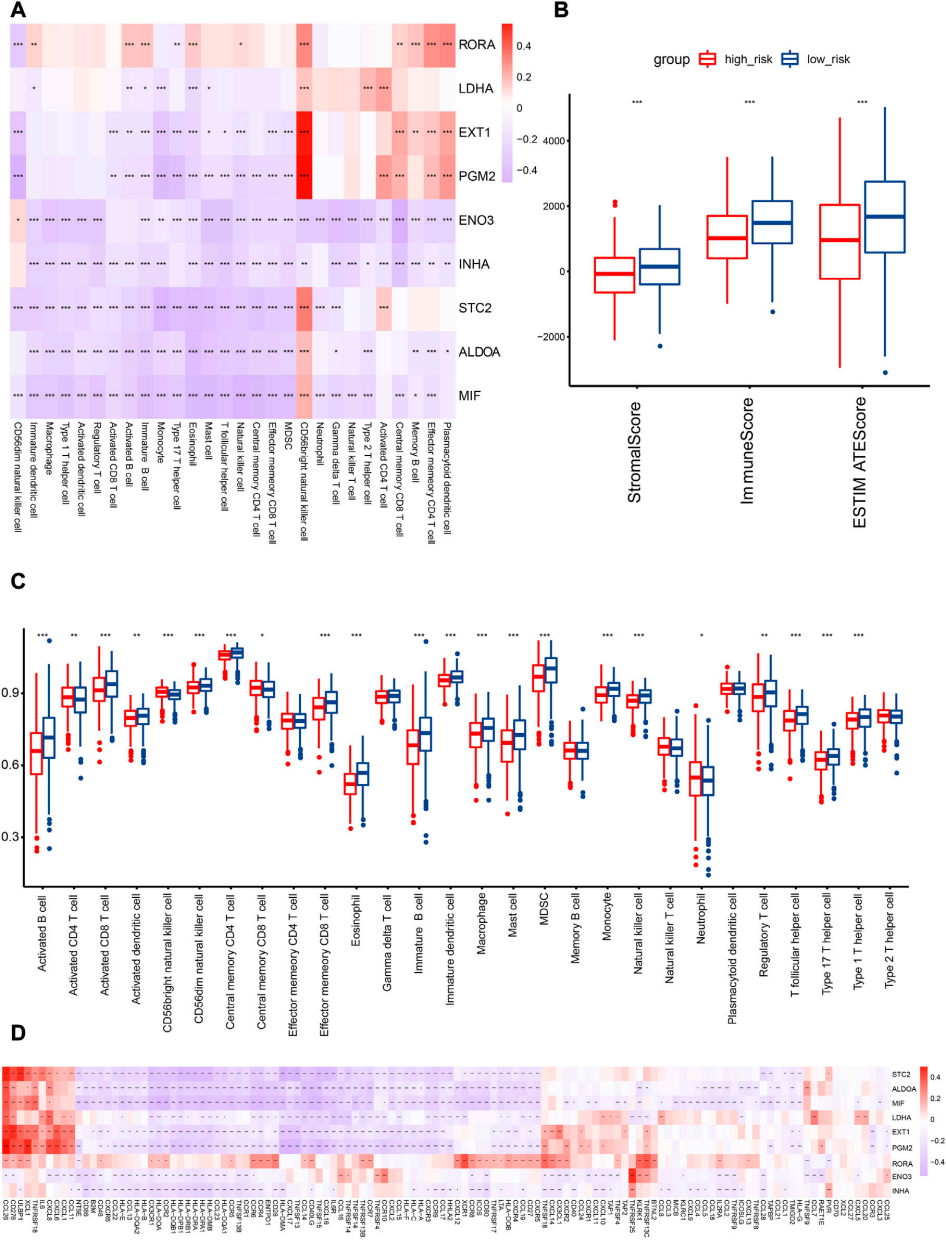
Association analysis of HRGs with TIME in the TCGA cohort. **(A)** Correlation analysis of HRGs in risk signature and immune cell abundance. **(B)** Comparison of immune score in the two subcategories. **(C)** Quantitative analysis of immune cell abundance between patients in the two risk categories. **(D)** HRGs and immunomodulator-related gene correlation analysis. Red represents a positive correlation, while blue represents a negative correlation in the heatmap. *p < 0.05; **p < 0.01; ***p < 0.001.

To further explore the risk score and immunity, we performed a correlation analysis of immune cell abundance and HRG expression in the risk model. The findings revealed an extensively significant negative correlation between immune cell abundance and HRGs, such as ENO3, INHA, and ALDOA. RORA was positively correlated with most immune cell types (Figure 8A).

In the TCGA cohort, immune cell abundance was calculated between the two risk categories. The abundance of activated CD8 T cells, activated B cells, effector memory CD8 T cells, and central memory CD4 T cells was higher in the low-risk category (Figure 8C). In addition, the immune score of the low-risk category was significantly greater than that of the high-risk category (Figure 8B).

To illustrate the relationship between the HRG model and the immune microenvironment, we generated the correlation pattern of the HRG model and immune-modulator-related genes. The results demonstrated that an extensively significant negative correlation existed, including STC2, ALDOA, MIF, LDHA, EXT1, PGM2, ENO3, and INHA. Only RORA was positively correlated with most immune modulators (Figure 8D).

The low-risk category was associated with high immune cell infiltration, immune-modulator-related gene expression, and immune scores, which are indicative of favorable clinical outcomes related to preexisting anticancer immunity.

### 3.8 Immune checkpoint expression between risk categories

**FIGURE 9:**
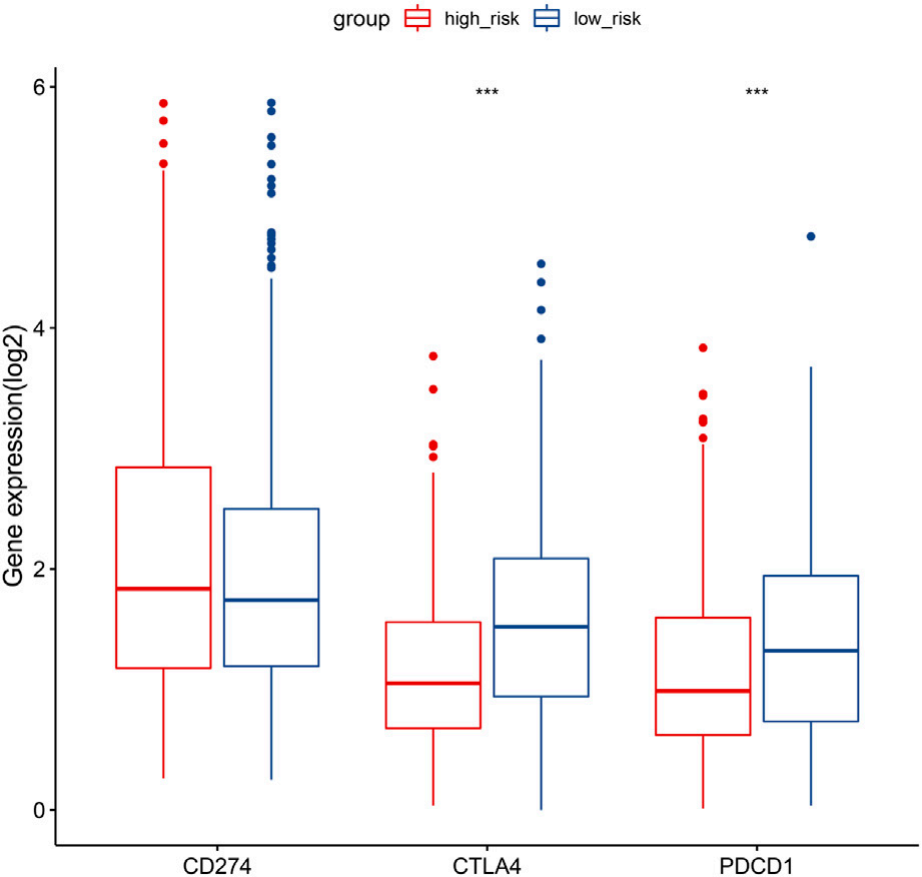
Gene expression comparison relating to immunological checkpoints in the two categories. Middle line of the box represents the median of the data, while the upper and lower limits of the box represent the upper and lower quartiles of the data, the line extending from the box represents 1.5 times the interquartile range (IQR) from the upper and lower quartiles. *p < 0.05; **p < 0.01; ***p < 0.001.

In consideration of the therapeutic significance of treatment approaches related to immune checkpoint blockade in NSCLC, the correlation between the risk score and several immune checkpoints, such as PD1, PDL1, and CTLA4, were analyzed. In the low-risk category, we found a substantial increase in the expression of PD1 and CTLA4, while the expression of PDL1 was not significantly different (Figure 9).

## 4 Discussion

NSCLC has a significant fatality rate and is one of the most frequently diagnosed cancers worldwide. Identifying accurate and effective signatures is of great importance for NSCLC prognostic prediction. Here, we constructed a novel HRG signature in a large NSCLC cohort, and training and validation datasets were used to confirm the signature’s efficacy.

Increasing evidence suggests that NSCLC patients who have intratumoral hypoxia have a worse prognosis and a more aggressive form of the disease (Carmeliet and Jain, 2011; Krock et al., 2011; Semenza, 2012; Eales et al., 2016; Mittal, 2018; Tirpe et al., 2019). Many monogenic studies of HRGs have been related to prognosis and tumor immunity in several cancers (Lee et al., 2019; Liu et al., 2020; Zhang et al., 2020). However, there has been no systematic investigation connecting TME features with the hypoxia-related signature in NSCLC. By combining data from many separate NSCLC studies, we were able to generate and verify a unique hypoxia risk signature. The established model confirmed that this signature could be used to effectively predict clinical outcomes and TME characteristics.

The most noticeable feature of malignant tumors is hypoxia. Prior studies have demonstrated that hypoxia contributes to the aggressive development of lung cancer (Smolle et al., 2020; Ouyang et al., 2021; Lane et al., 2022). However, due to its multifaceted nature, hypoxia's precise function in NSCLC progression is unclear. In the current research, we screened nine HRGs closely associated with NSCLC. Of these, four genes had been proven to be involved in tumor malignancy. ALDOA, an oncogene, has been identified as being involved in tumor cell malignant growth and worsening prognosis in hepatoma and pancreatic cancer (Ji et al., 2016). LDHA promotes papillary thyroid carcinoma metastasis by regulating EMT gene transcription (Hou et al., 2021). STC2 could promote the metastasis of HNSCC through the PI3K/AKT/Snail signaling pathway (Yang et al., 2017). In addition, EXT1 was demonstrated to participate in the process of cancer proliferation and migration by methylation regulation (Kong et al., 2021). Our results showed that this four-gene signature affecting the prognosis of NSCLC was incorporated into the risk model, suggesting that the established signature performed well in recurrence and prognosis prediction.

Hypoxia is a very important participant in the TME through various mechanisms. HRGs affect the infiltration of various immune cell subtypes which reshape the TIME. In particular, hypoxia enhances PD-L1 expression in multiple tumor cells through HIF-1’s direct binding to the HRE in the PDL1 promoter. The activity of T and NK cells is suppressed as a result of LDHA stimulation of the lactate metabolism (Brand et al., 2016). ALDOA has been shown to activate the NLRP3 inflammasome and induce the secretion of proinflammatory factors to regulate anticancer immune responses (Bai et al., 2022). RORA regulates ILC3s, macrophages, and Treg cells, which are crucial for ILC2 development (Haim-Vilmovsky et al., 2021). In addition, MIF regulates the inhibition of immune responses by enhancing harmful inflammation and eventually leads to the promotion of cancer metastasis (Sumaiya et al., 2022). In general, all the collective evidence inspired us to investigate the potential use of the hypoxia risk score to predict immunological features.

This study revealed a negative correlation between the risk score and the TIS and TIICs (such as activated B cells, activated CD8 T cells, central memory CD4 T cells, and effector memory CD8 T cells), which suggests that patients in the low-risk category had more preexisting anticancer immunity in their TME (Figure 8C). It is accepted that immune checkpoints prevent anticancer immunity in the TME (Nishino et al., 2017). Consistently, in the present study, the hypoxia risk score showed a negative correlation with the expression of several immune checkpoints, such as CTLA-4 and PD-1 (Figure 9). The TIS and immune checkpoint expression were considerably greater in low-risk category patients, indicating increased anticancer immunity and additional therapeutic targets in the TME. In other words, low-risk patients may gain more from ICB treatment, which may reactivate dormant tumoricidal immunity in the TME (Liu et al., 2021).

The innovative aspect of this study is its development of a prognostic model for NSCLC that can be used to predict the prognosis of both lung adenocarcinoma and lung squamous cell carcinoma without the need to distinguish specific cancer types. This approach potentially simplifies the diagnostic and treatment process for clinicians. Additionally, the ability to use this model to predict recurrence in NSCLC patients enhances its clinical utility. The study’s large sample size and good predictive performance in RNA-seq and microarray data platforms further highlight its strengths. Overall, the novel approach of using a prognostic model for different types of NSCLC represents a significant advancement in the field of cancer research. Nevertheless, this investigation had a few limitations. The risk score algorithm needs to be validated in real-world prospective cohort studies since the data used in the present research came from public sources. The sequencing techniques used for this research cohort varied and the accuracy of this formula might be affected to some extent. Additionally, the majority of patients were Caucasian, underscoring the need to investigate the risk score’s accuracy as a predictor of outcomes in patients of other races.

## 5 Conclusion

In total, a unique nine-gene risk score signature was established for NSCLC. The risk score was proven to correlate with OS, functional enrichment, the immune microenvironment, and therapy responsiveness. Additionally, it accurately predicted prognosis possibility depending on age, sex, and disease stage. These observations suggest that molecular risk stratification may aid in prognostic prediction and personalized precision therapy for NSCLC.

## Data Availability

The datasets presented in this study can be found in online repositories. The names of the repositories and accession number(s) can be found in the article/Supplementary Material.

## Abbreviation

AUC, area under the curve; HRGs, hypoxia-related genes; LASSO, least absolute shrinkage and selection operator; LUAD, lung adenocarcinoma; LUSC, lung squamous cell carcinoma; NSCLC, non-small cell lung cancer; ROC, receiver operating characteristic.
