# Supplementary material for: Novel hypoxia-related gene signature for predicting prognoses that correlate with the tumor immune microenvironment in NSCLC
Source: Front Genet. 2023 Apr 6;14:1115308. doi: 10.3389/fgene.2023.1115308 (PMC10115983; doi:10.3389/fgene.2023.1115308)
Supplement: Supplementary file 1 [file Table1.DOCX]

Supplementary Material


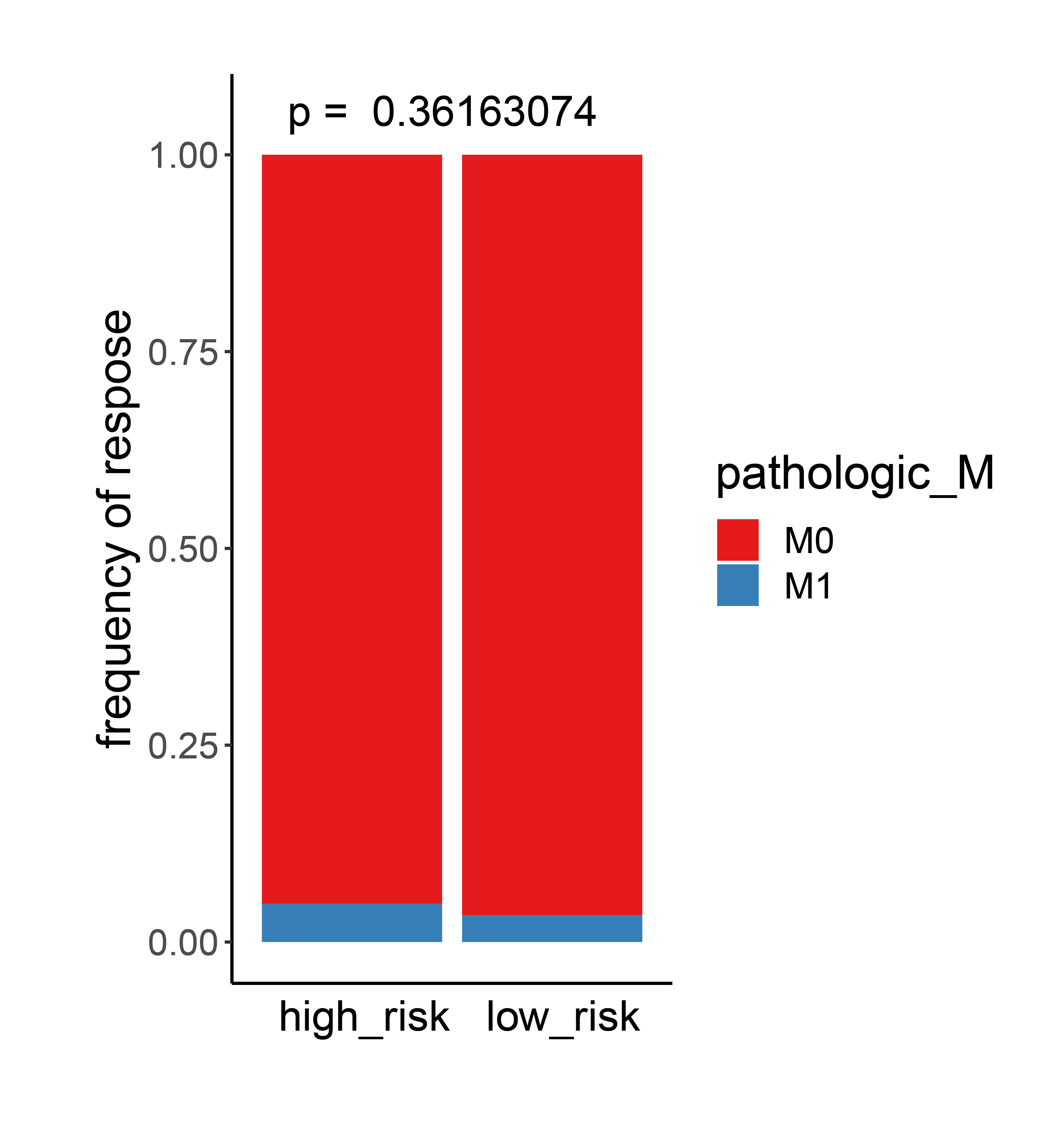


**Figure S1 Fisher test results of M stage between high and low risk categories in TCGA cohort.**


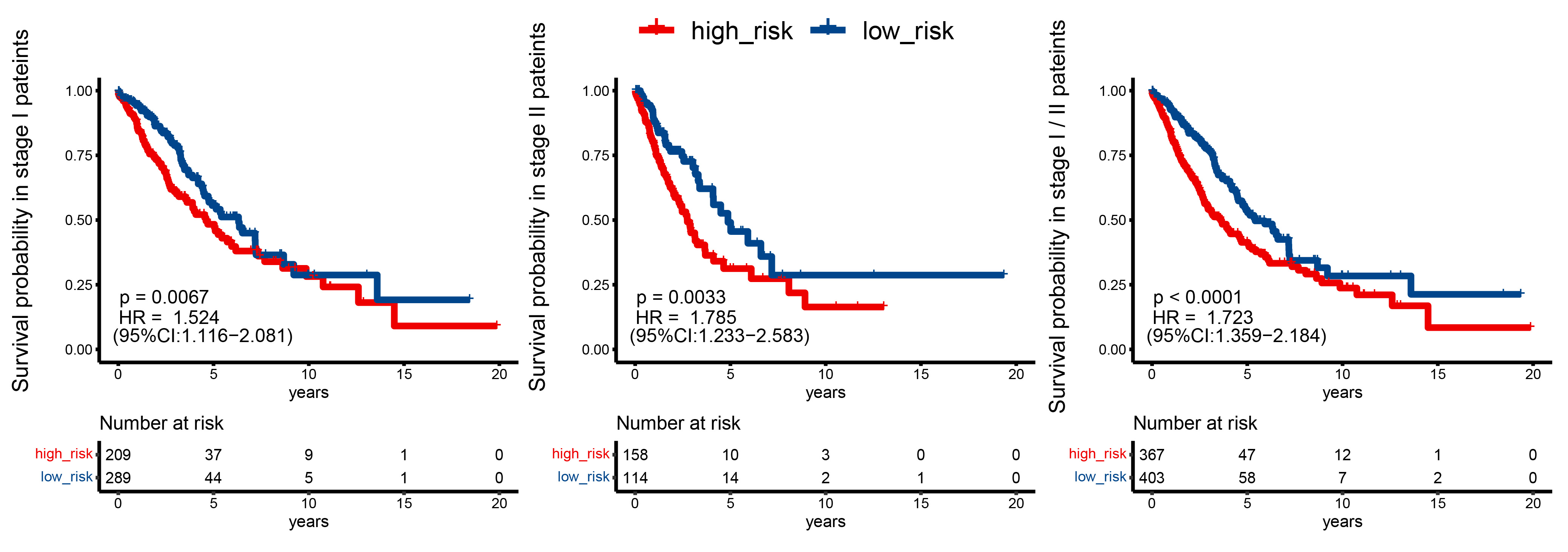


**Figure S2 Comparison of overall survival curves between two high-risk and low-risk categories among patients in TCGA early-stage NSCLC.**


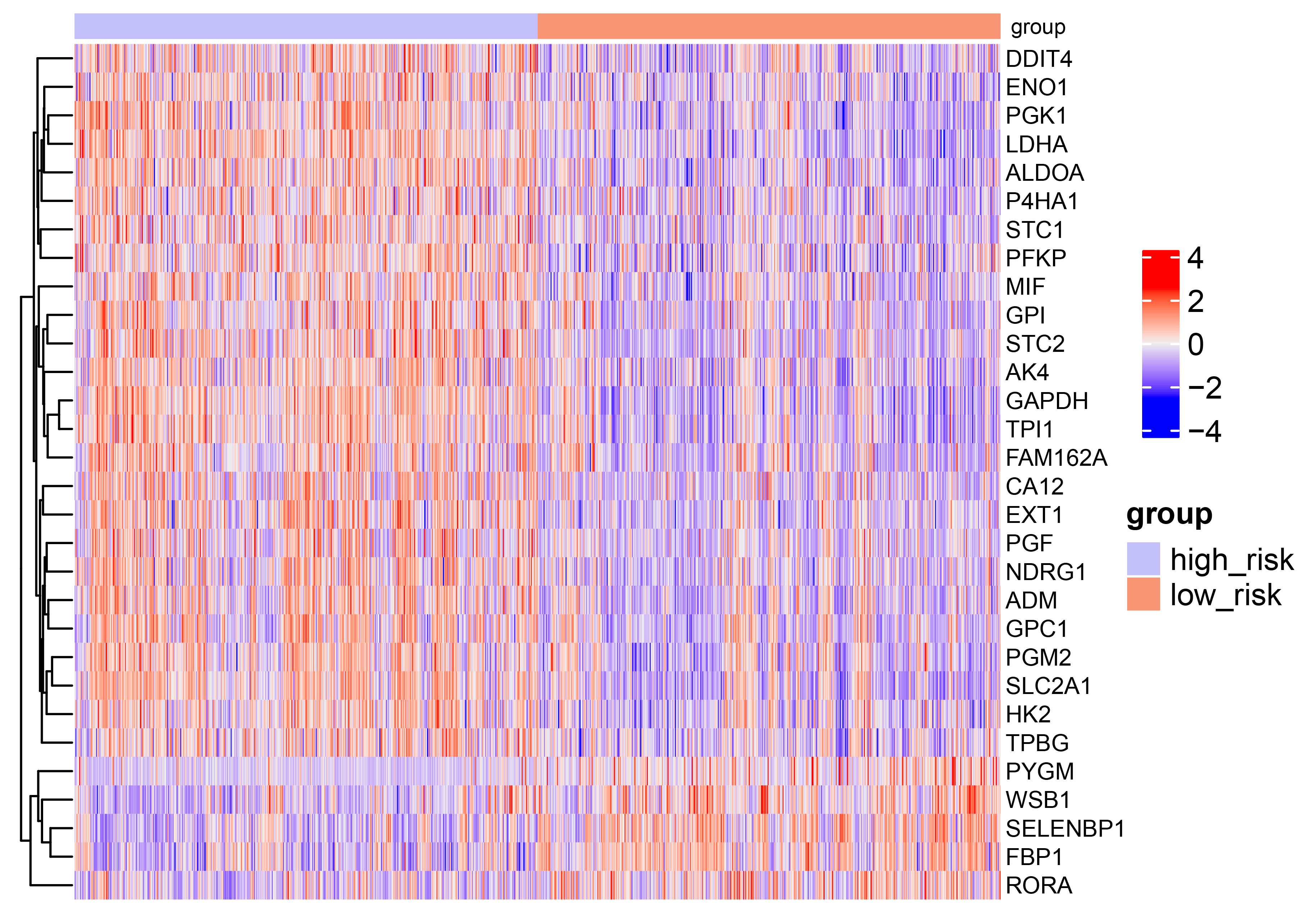


**Figure S3 Heat map of hypoxia-related gene expression in the TCGA cohort between high - and low-risk categories**

Table S1 Clinical information of NSCLS patient from GEO/TCGA in this study

| **Accession number** | **platform** | **Tumor** | **Normal** | **Survival** | **Stage** | **Gender** |
| --- | --- | --- | --- | --- | --- | --- |
| GSE30219 | GPL570 | 61 |  | 61 | 61 | 61 |
| GSE31210 | GPL570 | 226 |  | 226 | 226 | 226 |
| GSE37745 | GPL570 | 172 |  | 172 | 172 | 172 |
| GSE50081 | GPL570 | 169 |  | 169 | 169 | 169 |
| TCGA-LUAD/LUSC | Illumina | 1011 | 108 | 990 | 964 | 990 |

Table S2 Summary of Hypoxia Related Genes from MSigDB v7.4

| **Gene** | **Source** |  | **Gene** | **Source** |
| --- | --- | --- | --- | --- |
| PGK1 | HALLMARK_HYPOXIA |  | GALK1 | HALLMARK_HYPOXIA |
| PDK1 | HALLMARK_HYPOXIA |  | WSB1 | HALLMARK_HYPOXIA |
| GBE1 | HALLMARK_HYPOXIA |  | PYGM | HALLMARK_HYPOXIA |
| PFKL | HALLMARK_HYPOXIA |  | STC1 | HALLMARK_HYPOXIA |
| ALDOA | HALLMARK_HYPOXIA |  | ZNF292 | HALLMARK_HYPOXIA |
| ENO2 | HALLMARK_HYPOXIA |  | BTG1 | HALLMARK_HYPOXIA |
| PGM1 | HALLMARK_HYPOXIA |  | PLIN2 | HALLMARK_HYPOXIA |
| NDRG1 | HALLMARK_HYPOXIA |  | CSRP2 | HALLMARK_HYPOXIA |
| HK2 | HALLMARK_HYPOXIA |  | VLDLR | HALLMARK_HYPOXIA |
| ALDOC | HALLMARK_HYPOXIA |  | JMJD6 | HALLMARK_HYPOXIA |
| GPI | HALLMARK_HYPOXIA |  | EXT1 | HALLMARK_HYPOXIA |
| MXI1 | HALLMARK_HYPOXIA |  | F3 | HALLMARK_HYPOXIA |
| SLC2A1 | HALLMARK_HYPOXIA |  | PDK3 | HALLMARK_HYPOXIA |
| P4HA1 | HALLMARK_HYPOXIA |  | ANKZF1 | HALLMARK_HYPOXIA |
| ADM | HALLMARK_HYPOXIA |  | UGP2 | HALLMARK_HYPOXIA |
| P4HA2 | HALLMARK_HYPOXIA |  | ALDOB | HALLMARK_HYPOXIA |
| ENO1 | HALLMARK_HYPOXIA |  | STC2 | HALLMARK_HYPOXIA |
| PFKP | HALLMARK_HYPOXIA |  | ERRFI1 | HALLMARK_HYPOXIA |
| AK4 | HALLMARK_HYPOXIA |  | ENO3 | HALLMARK_HYPOXIA |
| FAM162A | HALLMARK_HYPOXIA |  | PNRC1 | HALLMARK_HYPOXIA |
| PFKFB3 | HALLMARK_HYPOXIA |  | HMOX1 | HALLMARK_HYPOXIA |
| VEGFA | HALLMARK_HYPOXIA |  | PGF | HALLMARK_HYPOXIA |
| BNIP3L | HALLMARK_HYPOXIA |  | GAPDHS | HALLMARK_HYPOXIA |
| TPI1 | HALLMARK_HYPOXIA |  | CHST2 | HALLMARK_HYPOXIA |
| KDM3A | HALLMARK_HYPOXIA |  | TMEM45A | HALLMARK_HYPOXIA |
| CCNG2 | HALLMARK_HYPOXIA |  | BCAN | HALLMARK_HYPOXIA |
| LDHA | HALLMARK_HYPOXIA |  | ATF3 | HALLMARK_HYPOXIA |
| GYS1 | HALLMARK_HYPOXIA |  | CAV1 | HALLMARK_HYPOXIA |
| GAPDH | HALLMARK_HYPOXIA |  | AMPD3 | HALLMARK_HYPOXIA |
| BHLHE40 | HALLMARK_HYPOXIA |  | GPC3 | HALLMARK_HYPOXIA |
| ANGPTL4 | HALLMARK_HYPOXIA |  | NDST1 | HALLMARK_HYPOXIA |
| JUN | HALLMARK_HYPOXIA |  | IRS2 | HALLMARK_HYPOXIA |
| SERPINE1 | HALLMARK_HYPOXIA |  | SAP30 | HALLMARK_HYPOXIA |
| LOX | HALLMARK_HYPOXIA |  | GAA | HALLMARK_HYPOXIA |
| GCK | HALLMARK_HYPOXIA |  | SDC4 | HALLMARK_HYPOXIA |
| PPFIA4 | HALLMARK_HYPOXIA |  | IER3 | HALLMARK_HYPOXIA |
| MAFF | HALLMARK_HYPOXIA |  | PKLR | HALLMARK_HYPOXIA |
| DDIT4 | HALLMARK_HYPOXIA |  | IGFBP1 | HALLMARK_HYPOXIA |
| SLC2A3 | HALLMARK_HYPOXIA |  | PLAUR | HALLMARK_HYPOXIA |
| IGFBP3 | HALLMARK_HYPOXIA |  | S100A4 | HALLMARK_HYPOXIA |
| NFIL3 | HALLMARK_HYPOXIA |  | RRAGD | HALLMARK_HYPOXIA |
| FOS | HALLMARK_HYPOXIA |  | ZFP36 | HALLMARK_HYPOXIA |
| RBPJ | HALLMARK_HYPOXIA |  | EGFR | HALLMARK_HYPOXIA |
| HK1 | HALLMARK_HYPOXIA |  | EDN2 | HALLMARK_HYPOXIA |
| CITED2 | HALLMARK_HYPOXIA |  | CDKN1A | HALLMARK_HYPOXIA |
| ISG20 | HALLMARK_HYPOXIA |  | RORA | HALLMARK_HYPOXIA |
| SDC2 | HALLMARK_HYPOXIA |  | TGFB3 | HALLMARK_HYPOXIA |
| MIF | HALLMARK_HYPOXIA |  | DUSP1 | HALLMARK_HYPOXIA |

**Table S2**. *(continued)*

| **Gene** | **Source** |  | **Gene** | **Source** |
| --- | --- | --- | --- | --- |
| PPP1R3C | HALLMARK_HYPOXIA |  | CASP6 | HALLMARK_HYPOXIA |
| DPYSL4 | HALLMARK_HYPOXIA |  | VHL | HALLMARK_HYPOXIA |
| KDELR3 | HALLMARK_HYPOXIA |  | FOXO3 | HALLMARK_HYPOXIA |
| DTNA | HALLMARK_HYPOXIA |  | PDGFB | HALLMARK_HYPOXIA |
| ADORA2B | HALLMARK_HYPOXIA |  | B3GALT6 | HALLMARK_HYPOXIA |
| HS3ST1 | HALLMARK_HYPOXIA |  | SLC2A5 | HALLMARK_HYPOXIA |
| NR3C1 | HALLMARK_HYPOXIA |  | SRPX | HALLMARK_HYPOXIA |
| KLF6 | HALLMARK_HYPOXIA |  | EFNA1 | HALLMARK_HYPOXIA |
| GPC4 | HALLMARK_HYPOXIA |  | GLRX | HALLMARK_HYPOXIA |
| TNFAIP3 | HALLMARK_HYPOXIA |  | ACKR3 | HALLMARK_HYPOXIA |
| CA12 | HALLMARK_HYPOXIA |  | PAM | HALLMARK_HYPOXIA |
| HEXA | HALLMARK_HYPOXIA |  | TGFBI | HALLMARK_HYPOXIA |
| BGN | HALLMARK_HYPOXIA |  | DCN | HALLMARK_HYPOXIA |
| PPP1R15A | HALLMARK_HYPOXIA |  | SIAH2 | HALLMARK_HYPOXIA |
| PGM2 | HALLMARK_HYPOXIA |  | PLAC8 | HALLMARK_HYPOXIA |
| PIM1 | HALLMARK_HYPOXIA |  | FBP1 | HALLMARK_HYPOXIA |
| PRDX5 | HALLMARK_HYPOXIA |  | TPST2 | HALLMARK_HYPOXIA |
| NAGK | HALLMARK_HYPOXIA |  | PHKG1 | HALLMARK_HYPOXIA |
| CDKN1B | HALLMARK_HYPOXIA |  | MYH9 | HALLMARK_HYPOXIA |
| BRS3 | HALLMARK_HYPOXIA |  | CDKN1C | HALLMARK_HYPOXIA |
| TKTL1 | HALLMARK_HYPOXIA |  | GRHPR | HALLMARK_HYPOXIA |
| MT1E | HALLMARK_HYPOXIA |  | PCK1 | HALLMARK_HYPOXIA |
| ATP7A | HALLMARK_HYPOXIA |  | INHA | HALLMARK_HYPOXIA |
| MT2A | HALLMARK_HYPOXIA |  | HSPA5 | HALLMARK_HYPOXIA |
| SDC3 | HALLMARK_HYPOXIA |  | NDST2 | HALLMARK_HYPOXIA |
| TIPARP | HALLMARK_HYPOXIA |  | NEDD4L | HALLMARK_HYPOXIA |
| PKP1 | HALLMARK_HYPOXIA |  | TPBG | HALLMARK_HYPOXIA |
| ANXA2 | HALLMARK_HYPOXIA |  | XPNPEP1 | HALLMARK_HYPOXIA |
| PGAM2 | HALLMARK_HYPOXIA |  | IL6 | HALLMARK_HYPOXIA |
| DDIT3 | HALLMARK_HYPOXIA |  | SLC6A6 | HALLMARK_HYPOXIA |
| PRKCA | HALLMARK_HYPOXIA |  | MAP3K1 | HALLMARK_HYPOXIA |
| SLC37A4 | HALLMARK_HYPOXIA |  | LDHC | HALLMARK_HYPOXIA |
| CXCR4 | HALLMARK_HYPOXIA |  | AKAP12 | HALLMARK_HYPOXIA |
| EFNA3 | HALLMARK_HYPOXIA |  | TES | HALLMARK_HYPOXIA |
| CP | HALLMARK_HYPOXIA |  | KIF5A | HALLMARK_HYPOXIA |
| KLF7 | HALLMARK_HYPOXIA |  | LALBA | HALLMARK_HYPOXIA |
| CHST3 | HALLMARK_HYPOXIA |  | COL5A1 | HALLMARK_HYPOXIA |
| TPD52 | HALLMARK_HYPOXIA |  | GPC1 | HALLMARK_HYPOXIA |
| LXN | HALLMARK_HYPOXIA |  | HDLBP | HALLMARK_HYPOXIA |
| B4GALNT2 | HALLMARK_HYPOXIA |  | ILVBL | HALLMARK_HYPOXIA |
| PPARGC1A | HALLMARK_HYPOXIA |  | NCAN | HALLMARK_HYPOXIA |
| BCL2 | HALLMARK_HYPOXIA |  | TGM2 | HALLMARK_HYPOXIA |
| GCNT2 | HALLMARK_HYPOXIA |  | ETS1 | HALLMARK_HYPOXIA |
| HAS1 | HALLMARK_HYPOXIA |  | HOXB9 | HALLMARK_HYPOXIA |
| KLHL24 | HALLMARK_HYPOXIA |  | SELENBP1 | HALLMARK_HYPOXIA |
| SCARB1 | HALLMARK_HYPOXIA |  | FOSL2 | HALLMARK_HYPOXIA |
| SLC25A1 | HALLMARK_HYPOXIA |  | SULT2B1 | HALLMARK_HYPOXIA |

Table S3 differential expression results of Hypoxia related genes between normal and tumor group in TCGA cohort

| **gene** | **logFC** | **AveExpr** | **t** | **P.Value** | **adj.P.Val** |
| --- | --- | --- | --- | --- | --- |
| PGK1 | 0.997024418 | 6.958999984 | 13.09796598 | 1.43E-36 | 6.30E-36 |
| PDK1 | 1.118557064 | 1.814528023 | 17.09298552 | 2.24E-58 | 2.36E-57 |
| PFKL | 0.474525235 | 4.433057635 | 8.931134343 | 1.70E-18 | 4.24E-18 |
| ALDOA | 1.316805886 | 7.786000969 | 20.76843584 | 2.72E-81 | 5.75E-80 |
| ENO2 | 0.807770261 | 4.079166949 | 7.83596921 | 1.08E-14 | 2.41E-14 |
| PGM1 | -0.676293848 | 4.343499439 | -10.4540849 | 1.80E-24 | 5.53E-24 |
| NDRG1 | 0.990707229 | 6.201270863 | 7.485678434 | 1.44E-13 | 3.07E-13 |
| HK2 | 0.277612206 | 4.184145038 | 2.33459096 | 0.01974101 | 0.0242224 |
| ALDOC | 0.955870828 | 3.640935007 | 8.328360435 | 2.37E-16 | 5.63E-16 |
| GPI | 1.488021012 | 5.622449968 | 18.09461733 | 2.17E-64 | 2.95E-63 |
| SLC2A1 | 3.921713779 | 5.595302123 | 21.3376057 | 5.01E-85 | 1.59E-83 |
| P4HA1 | 0.911960964 | 5.12427435 | 12.31627282 | 8.85E-33 | 3.58E-32 |
| ADM | 0.818676419 | 3.684007999 | 5.176328994 | 2.68E-07 | 4.28E-07 |
| ENO1 | 1.300805779 | 8.835268997 | 21.02569319 | 5.65E-83 | 1.53E-81 |
| PFKP | 1.772904108 | 4.750186586 | 18.91446974 | 1.90E-69 | 3.00E-68 |
| AK4 | 1.632836895 | 2.163003831 | 16.63746425 | 1.05E-55 | 9.46E-55 |
| FAM162A | 1.140395752 | 4.067522627 | 12.56098084 | 6.00E-34 | 2.48E-33 |
| PFKFB3 | -0.531083753 | 4.521093572 | -6.15383336 | 1.05E-09 | 1.83E-09 |
| BNIP3L | -0.303271231 | 4.945967955 | -5.22275529 | 2.10E-07 | 3.38E-07 |
| TPI1 | 1.450425357 | 7.846017006 | 20.78160251 | 2.23E-81 | 5.31E-80 |
| KDM3A | 0.56792463 | 2.757906411 | 10.27927173 | 9.57E-24 | 2.75E-23 |
| CCNG2 | 0.147230818 | 2.806665728 | 2.326731574 | 0.0201574 | 0.0245507 |
| LDHA | 1.38634422 | 6.968379501 | 19.89905976 | 1.13E-75 | 1.95E-74 |
| GYS1 | 0.631216042 | 4.078993956 | 10.40099301 | 3.00E-24 | 9.05E-24 |
| GAPDH | 2.097077517 | 9.74509293 | 22.7268212 | 2.58E-94 | 1.63E-92 |
| ANGPTL4 | 0.899359287 | 3.3839604 | 6.005703733 | 2.57E-09 | 4.33E-09 |
| JUN | -1.02578843 | 5.79015991 | -11.372204 | 1.96E-28 | 7.01E-28 |
| LOX | 0.283395603 | 2.930142159 | 2.741219137 | 0.00621869 | 0.0080928 |
| GCK | 0.057913296 | 0.163917445 | 2.011744082 | 0.04448632 | 0.0531598 |
| PPFIA4 | 0.602556835 | 0.698275981 | 10.57262972 | 5.74E-25 | 1.79E-24 |
| MAFF | -0.974330708 | 2.796450099 | -14.118705 | 8.95E-42 | 4.86E-41 |
| DDIT4 | 1.209263157 | 5.832123378 | 9.617259242 | 4.31E-21 | 1.15E-20 |
| SLC2A3 | -1.266222483 | 3.361053636 | -11.1882814 | 1.28E-27 | 4.50E-27 |
| IGFBP3 | 2.11299372 | 5.820342327 | 14.30714451 | 9.15E-43 | 5.43E-42 |
| NFIL3 | -0.427776229 | 4.292763229 | -6.31102601 | 3.99E-10 | 7.15E-10 |
| FOS | -2.02214494 | 5.960653974 | -14.2889174 | 1.14E-42 | 6.38E-42 |
| RBPJ | -0.122429485 | 3.491163083 | -2.33422156 | 0.01976041 | 0.0242224 |
| HK1 | 0.299598563 | 4.926071352 | 4.665528432 | 3.45E-06 | 5.24E-06 |

**Table S3.***(continued)*

| **gene** | **logFC** | **AveExpr** | **t** | **P.Value** | **adj.P.Val** |
| --- | --- | --- | --- | --- | --- |
| CDKN1A | -0.61309 | 5.501597 | -7.16639 | 1.39E-12 | 2.79E-12 |
| RORA | -0.5613 | 1.234772 | -10.7019 | 1.63E-25 | 5.43E-25 |
| DUSP1 | -2.29456 | 6.611562 | -17.9268 | 2.28E-63 | 2.71E-62 |
| MIF | 1.609548 | 5.473013 | 17.97835 | 1.11E-63 | 1.40E-62 |
| PPP1R3C | -0.75707 | 1.835666 | -7.85587 | 9.27E-15 | 2.10E-14 |
| DPYSL4 | 0.579076 | 0.944443 | 6.494326 | 1.25E-10 | 2.38E-10 |
| KDELR3 | 1.509414 | 4.130946 | 15.7919 | 7.22E-51 | 5.08E-50 |
| DTNA | -0.19968 | 0.87396 | -3.37258 | 0.00077 | 0.0011 |
| ADORA2B | 0.660052 | 2.355742 | 7.909512 | 6.17E-15 | 1.41E-14 |
| HS3ST1 | 0.638276 | 1.557211 | 7.251718 | 7.67E-13 | 1.55E-12 |
| NR3C1 | -0.71544 | 3.226452 | -12.5638 | 5.82E-34 | 2.46E-33 |
| KLF6 | -1.83949 | 4.707593 | -22.7788 | 1.15E-94 | 1.09E-92 |
| GPC4 | -0.376 | 4.030957 | -3.18884 | 0.001468 | 0.002007 |
| TNFAIP3 | -0.79634 | 3.833126 | -7.7811 | 1.63E-14 | 3.60E-14 |
| CA12 | 1.305843 | 2.792763 | 7.393889 | 2.79E-13 | 5.82E-13 |
| HEXA | -0.17737 | 3.047728 | -3.70795 | 0.000219 | 0.000316 |
| PPP1R15A | -1.62339 | 4.904285 | -21.5472 | 2.06E-86 | 7.82E-85 |
| PGM2 | 0.659846 | 3.476657 | 7.640826 | 4.62E-14 | 9.98E-14 |
| NAGK | 0.272749 | 3.077069 | 6.017728 | 2.39E-09 | 4.06E-09 |
| MT1E | -1.08448 | 4.420351 | -7.25718 | 7.38E-13 | 1.51E-12 |
| MT2A | -0.72554 | 6.355867 | -5.45193 | 6.13E-08 | 1.01E-07 |
| SDC3 | -0.19937 | 3.824351 | -2.50181 | 0.012498 | 0.016154 |
| TIPARP | -0.91895 | 3.657864 | -10.0637 | 7.25E-23 | 2.06E-22 |
| PKP1 | 2.778677 | 3.712448 | 9.319135 | 6.05E-20 | 1.60E-19 |
| ANXA2 | -0.16734 | 6.806146 | -2.1365 | 0.032856 | 0.03951 |
| DDIT3 | 0.743575 | 4.170176 | 8.666107 | 1.54E-17 | 3.76E-17 |
| PRKCA | -0.21659 | 1.730518 | -2.84224 | 0.004561 | 0.006018 |
| SLC37A4 | 0.672044 | 2.59358 | 14.29274 | 1.09E-42 | 6.28E-42 |
| CXCR4 | -0.32768 | 5.027064 | -2.96432 | 0.003098 | 0.004116 |
| EFNA3 | 1.76492 | 2.036979 | 20.63804 | 1.93E-80 | 3.66E-79 |
| CP | 1.784175 | 3.858617 | 9.239071 | 1.21E-19 | 3.16E-19 |
| KLF7 | -0.39686 | 2.380428 | -6.19938 | 7.96E-10 | 1.41E-09 |
| CHST3 | 0.234812 | 2.407884 | 2.387582 | 0.017125 | 0.021406 |
| TPD52 | 1.032528 | 3.772937 | 14.48452 | 1.05E-43 | 6.44E-43 |
| LXN | -0.40629 | 3.484008 | -4.85968 | 1.34E-06 | 2.07E-06 |
| B4GALNT2 | 0.380982 | 0.44327 | 4.877398 | 1.23E-06 | 1.92E-06 |
| PPARGC1A | -0.5859 | 0.625841 | -9.00042 | 9.43E-19 | 2.42E-18 |
| GCNT2 | -0.22888 | 2.00532 | -3.20956 | 0.001367 | 0.001882 |

**Table S3.** *(continued)*

| **gene** | **logFC** | **AveExpr** | **t** | **P.Value** | **adj.P.Val** |
| --- | --- | --- | --- | --- | --- |
| CITED2 | -1.753839657 | 4.856376757 | -15.5591403 | 1.45E-49 | 9.53E-49 |
| GALK1 | 0.325658249 | 3.032434898 | 5.476862739 | 5.34E-08 | 8.91E-08 |
| WSB1 | -0.250241539 | 4.32392468 | -2.4945507 | 0.01275506 | 0.0163747 |
| PYGM | -0.410844792 | 0.309017573 | -16.9787175 | 1.06E-57 | 1.06E-56 |
| ZNF292 | 0.304203847 | 1.967011809 | 6.330995573 | 3.52E-10 | 6.37E-10 |
| BTG1 | -0.177622287 | 5.298544111 | -2.49183125 | 0.01285257 | 0.0163892 |
| PLIN2 | -1.041329948 | 3.672082517 | -9.92589883 | 2.60E-22 | 7.06E-22 |
| CSRP2 | 0.456107471 | 2.002944101 | 4.788332296 | 1.91E-06 | 2.92E-06 |
| VLDLR | -0.350460429 | 1.79593861 | -4.60185389 | 4.67E-06 | 6.98E-06 |
| JMJD6 | 0.20083657 | 2.55361031 | 4.913976806 | 1.03E-06 | 1.61E-06 |
| EXT1 | 0.27648552 | 3.43751361 | 4.050985016 | 5.45E-05 | 8.03E-05 |
| F3 | -0.732090888 | 4.929953899 | -5.26937574 | 1.64E-07 | 2.67E-07 |
| PDK3 | 0.419344083 | 2.298667229 | 6.339642368 | 3.33E-10 | 6.09E-10 |
| ANKZF1 | 0.770041101 | 2.86874404 | 13.63363059 | 2.87E-39 | 1.48E-38 |
| UGP2 | 0.194138456 | 4.326716587 | 4.153632969 | 3.52E-05 | 5.23E-05 |
| STC2 | 0.791629907 | 2.236976872 | 6.445955523 | 1.70E-10 | 3.15E-10 |
| ENO3 | 0.610417874 | 1.207170525 | 6.515862567 | 1.09E-10 | 2.11E-10 |
| PNRC1 | -0.665092827 | 5.003952289 | -11.9990191 | 2.73E-31 | 1.04E-30 |
| HMOX1 | -0.866870967 | 4.596987253 | -8.14690532 | 9.91E-16 | 2.32E-15 |
| PGF | 1.085630542 | 1.869463121 | 10.68296489 | 1.96E-25 | 6.43E-25 |
| GAPDHS | 0.020799466 | 0.029118174 | 3.167731646 | 0.00157792 | 0.0021415 |
| CHST2 | 0.629024386 | 2.672715043 | 4.981043684 | 7.32E-07 | 1.16E-06 |
| TMEM45A | 0.777143093 | 2.550371413 | 6.062986639 | 1.82E-09 | 3.12E-09 |
| BCAN | 0.362017102 | 0.409933373 | 6.469587736 | 1.47E-10 | 2.76E-10 |
| ATF3 | -1.545121181 | 3.689486583 | -13.2364308 | 2.92E-37 | 1.35E-36 |
| CAV1 | -3.700086818 | 4.921292472 | -29.8425565 | 1.97E-144 | 3.74E-142 |
| AMPD3 | 0.474398951 | 1.982217037 | 8.68703326 | 1.30E-17 | 3.21E-17 |
| GPC3 | -1.911285899 | 3.833292023 | -9.93814262 | 2.32E-22 | 6.40E-22 |
| NDST1 | -1.376133455 | 3.586075946 | -22.1735306 | 1.37E-90 | 6.53E-89 |
| IRS2 | -0.481861806 | 2.534582776 | -4.65231874 | 3.67E-06 | 5.54E-06 |
| SAP30 | 0.412477903 | 2.627550443 | 7.120628306 | 1.92E-12 | 3.80E-12 |
| GAA | -0.189652651 | 5.134571109 | -2.46019856 | 0.01403634 | 0.0176616 |
| SDC4 | -1.022041472 | 7.15603948 | -10.3598085 | 4.45E-24 | 1.30E-23 |
| IER3 | 0.910064424 | 5.674217949 | 7.743928863 | 2.15E-14 | 4.69E-14 |
| PKLR | 0.033761183 | 0.038725731 | 2.763288075 | 0.00581607 | 0.0076211 |
| S100A4 | -1.932763985 | 6.660436809 | -16.3478412 | 4.94E-54 | 3.91E-53 |
| RRAGD | -0.490216475 | 2.596373286 | -6.09107725 | 1.54E-09 | 2.66E-09 |
| ZFP36 | -1.983024904 | 6.676365883 | -17.49984 | 8.52E-61 | 9.53E-60 |
| EGFR | 0.421290172 | 3.84447225 | 3.295795463 | 0.00101224 | 0.0014188 |
| EDN2 | 0.910873188 | 1.686333829 | 6.503247898 | 1.18E-10 | 2.27E-10 |
| SELENBP1 | -2.766853464 | 4.369248035 | -15.6168408 | 6.93E-50 | 4.70E-49 |
| SULT2B1 | -0.605883597 | 2.157444879 | -5.3998328 | 8.14E-08 | 1.33E-07 |

**Table S3.** *(continued)*

| **gene** | **logFC** | **AveExpr** | **t** | **P.Value** | **adj.P.Val** |
| --- | --- | --- | --- | --- | --- |
| HAS1 | -0.90429 | 0.477111 | -13.3189 | 1.13E-37 | 5.35E-37 |
| KLHL24 | 0.256413 | 3.357092 | 3.224082 | 0.0013 | 0.001803 |
| SLC25A1 | 0.676938 | 5.382111 | 10.66816 | 2.27E-25 | 7.30E-25 |
| SDC2 | -1.04741 | 3.510073 | -10.0026 | 1.28E-22 | 3.58E-22 |
| CASP6 | 0.686303 | 3.476395 | 11.67805 | 8.18E-30 | 2.99E-29 |
| VHL | 0.367515 | 3.258379 | 8.041648 | 2.24E-15 | 5.19E-15 |
| FOXO3 | -0.49888 | 3.086293 | -8.33925 | 2.17E-16 | 5.23E-16 |
| PDGFB | -1.28917 | 2.889873 | -16.0945 | 1.39E-52 | 1.06E-51 |
| B3GALT6 | 0.339194 | 3.292558 | 6.521998 | 1.05E-10 | 2.05E-10 |
| SLC2A5 | 0.867779 | 1.127577 | 13.1417 | 8.65E-37 | 3.91E-36 |
| SRPX | -1.72214 | 2.374176 | -16.569 | 2.61E-55 | 2.16E-54 |
| GLRX | -0.89584 | 2.365119 | -10.5943 | 4.66E-25 | 1.47E-24 |
| ACKR3 | 0.541636 | 4.345437 | 2.969251 | 0.003049 | 0.00408 |
| PAM | -0.28081 | 4.277263 | -3.35004 | 0.000835 | 0.001184 |
| TGFBI | 0.304755 | 4.808847 | 2.379521 | 0.017502 | 0.021735 |
| DCN | -1.70733 | 4.796708 | -13.6588 | 2.14E-39 | 1.13E-38 |
| SIAH2 | 1.114799 | 4.248817 | 12.11835 | 7.57E-32 | 2.94E-31 |
| PLAC8 | -1.37293 | 2.119707 | -10.9215 | 1.86E-26 | 6.44E-26 |
| FBP1 | -2.54365 | 4.944307 | -16.6063 | 1.59E-55 | 1.37E-54 |
| TPST2 | -0.38253 | 2.887797 | -7.41954 | 2.32E-13 | 4.90E-13 |
| PHKG1 | -0.0705 | 0.501399 | -2.48583 | 0.01307 | 0.016555 |
| MYH9 | -0.23492 | 7.263498 | -3.00297 | 0.002733 | 0.003683 |
| CDKN1C | -1.06371 | 2.548978 | -12.1991 | 3.16E-32 | 1.25E-31 |
| GRHPR | 0.152053 | 3.345153 | 3.294863 | 0.001016 | 0.001419 |
| INHA | 0.802559 | 0.845623 | 6.190102 | 8.43E-10 | 1.48E-09 |
| HSPA5 | 0.849965 | 7.762761 | 16.95765 | 1.40E-57 | 1.33E-56 |
| NEDD4L | -0.96062 | 2.836293 | -13.3844 | 5.28E-38 | 2.57E-37 |
| TPBG | 1.528656 | 2.889517 | 18.66058 | 7.19E-68 | 1.05E-66 |
| XPNPEP1 | 0.469208 | 3.276939 | 10.72856 | 1.25E-25 | 4.26E-25 |
| IL6 | -1.89331 | 2.220174 | -13.5531 | 7.39E-39 | 3.70E-38 |
| SLC6A6 | -0.1452 | 3.607664 | -1.97542 | 0.048466 | 0.057554 |
| LDHC | 0.169756 | 0.319825 | 3.727904 | 0.000203 | 0.000294 |
| AKAP12 | -1.67489 | 2.143908 | -14.9401 | 3.73E-46 | 2.36E-45 |
| KIF5A | 0.293838 | 0.33008 | 6.44581 | 1.71E-10 | 3.15E-10 |
| COL5A1 | 1.543468 | 4.435259 | 11.69803 | 6.64E-30 | 2.47E-29 |
| GPC1 | 1.45084 | 4.296158 | 8.978908 | 1.13E-18 | 2.87E-18 |
| HDLBP | 0.521735 | 5.773253 | 10.37029 | 4.02E-24 | 1.19E-23 |
| ILVBL | 0.219542 | 3.476978 | 4.014609 | 6.35E-05 | 9.28E-05 |
| NCAN | 0.030399 | 0.030373 | 2.183363 | 0.029216 | 0.035357 |
| TGM2 | -1.67634 | 5.261683 | -12.8615 | 2.09E-35 | 9.01E-35 |
| ETS1 | -1.47108 | 3.761658 | -16.0179 | 3.81E-52 | 2.78E-51 |
| HOXB9 | 1.021663 | 0.957995 | 7.335795 | 4.22E-13 | 8.72E-13 |

**Table S4** Univariate Cox regression analysis results of Hypoxia related genes in TCGA cohort

| **gene** | **coef** | **exp(coef)** | **pvalue** | **lower .95** | **upper .95** |
| --- | --- | --- | --- | --- | --- |
| ALDOA | 0.175900855 | 1.19231984 | 0.00205208 | 1.06615638 | 1.33341283 |
| PGM1 | 0.105379248 | 1.111131925 | 0.04549909 | 1.00211227 | 1.232011814 |
| SLC2A1 | 0.18137604 | 1.198865916 | 0.00199623 | 1.06861145 | 1.344997275 |
| P4HA1 | 0.146516123 | 1.157793598 | 0.00369456 | 1.04875102 | 1.278173747 |
| ADM | 0.110611066 | 1.116960399 | 0.02276094 | 1.01553802 | 1.228511891 |
| ENO1 | 0.139568164 | 1.149777176 | 0.02039332 | 1.02184525 | 1.293725788 |
| PFKP | 0.195952473 | 1.216469088 | 0.00072388 | 1.08582253 | 1.362835088 |
| TPI1 | 0.115737357 | 1.122700964 | 0.04214365 | 1.00411686 | 1.255289601 |
| LDHA | 0.265427538 | 1.303988362 | 7.57E-06 | 1.16093316 | 1.464671445 |
| GAPDH | 0.160898947 | 1.174566269 | 0.00588055 | 1.04749947 | 1.317046895 |
| ANGPTL4 | 0.175659057 | 1.192031575 | 0.00055291 | 1.07893421 | 1.316984165 |
| LOX | 0.124470515 | 1.132548627 | 0.00782878 | 1.03327692 | 1.241357825 |
| MAFF | 0.135907013 | 1.145575365 | 0.01884637 | 1.02274191 | 1.283161375 |
| DDIT4 | 0.139960141 | 1.150227952 | 0.00697278 | 1.03903389 | 1.273321649 |
| PLIN2 | 0.093216433 | 1.097699288 | 0.04999651 | 1.00000142 | 1.204942017 |
| EXT1 | 0.1391796 | 1.149330501 | 0.0030472 | 1.04824059 | 1.260169285 |
| STC2 | 0.153569125 | 1.165988383 | 0.00179442 | 1.05883491 | 1.283985721 |
| ENO3 | -0.12209944 | 0.885060355 | 0.02218327 | 0.79713671 | 0.982681913 |
| TMEM45A | 0.098734982 | 1.103773741 | 0.03574167 | 1.00659931 | 1.210329137 |
| BCAN | 0.079996796 | 1.083283597 | 0.03555432 | 1.00541974 | 1.16717755 |
| CAV1 | 0.162650335 | 1.176625193 | 0.01190738 | 1.03654096 | 1.335641231 |
| IER3 | 0.138650925 | 1.148723039 | 0.00602003 | 1.04051242 | 1.268187288 |
| CDKN1A | 0.172844779 | 1.188681583 | 0.00231354 | 1.06359342 | 1.328481245 |
| RORA | -0.11831981 | 0.88841188 | 0.02477575 | 0.80121766 | 0.985095202 |
| MIF | 0.12912379 | 1.137830967 | 0.01911027 | 1.02135122 | 1.267594617 |
| GPC4 | -0.11257014 | 0.893534676 | 0.02293785 | 0.8109292 | 0.984554777 |
| PGM2 | 0.134030655 | 1.143427871 | 0.00629431 | 1.0386037 | 1.258831733 |
| MT2A | 0.173091038 | 1.188974342 | 0.00070447 | 1.07567962 | 1.314201704 |
| ANXA2 | 0.147179855 | 1.158562317 | 0.00439406 | 1.0469762 | 1.282041214 |
| PRKCA | 0.099183582 | 1.104269005 | 0.04276582 | 1.00323757 | 1.215474854 |
| HAS1 | 0.12872601 | 1.137378451 | 0.02981108 | 1.01267787 | 1.277434593 |
| PDGFB | 0.178564258 | 1.195499702 | 0.00095302 | 1.07534291 | 1.329082582 |
| TGFBI | 0.095942457 | 1.100695725 | 0.03974837 | 1.00450738 | 1.206094757 |
| INHA | 0.114391037 | 1.121190466 | 0.01282468 | 1.02459697 | 1.22689028 |
| HSPA5 | 0.11115551 | 1.117568686 | 0.04553008 | 1.00221252 | 1.24620252 |
| TPBG | 0.122641946 | 1.130479576 | 0.02969709 | 1.01215313 | 1.262639061 |
| AKAP12 | 0.227723785 | 1.255738423 | 6.49E-06 | 1.13741344 | 1.386372742 |
| COL5A1 | 0.152456087 | 1.164691316 | 0.00300128 | 1.05312934 | 1.288071477 |
| TGM2 | 0.110671695 | 1.117028121 | 0.03366058 | 1.00858932 | 1.237125749 |
| SELENBP1 | -0.10423492 | 0.901013605 | 0.04788853 | 0.81261259 | 0.999031423 |
| SULT2B1 | 0.104888964 | 1.110587288 | 0.0330524 | 1.00847438 | 1.223039616 |
